# Relating Design and Optoelectronic Properties of 1,4-Dihydropyrrolo[3,2-*b*]pyrroles Bearing Biphenyl Substituents

**DOI:** 10.1021/acs.jpcb.3c03061

**Published:** 2023-08-10

**Authors:** Allison M. Hawks, Drake Altman, Ryan Faddis, Ethan M. Wagner, Kenneth-John J. Bell, Ariane Charland-Martin, Graham S. Collier

**Affiliations:** Department of Chemistry and Biochemistry, Kennesaw State University, Kennesaw, Georgia 30144, United States

## Abstract

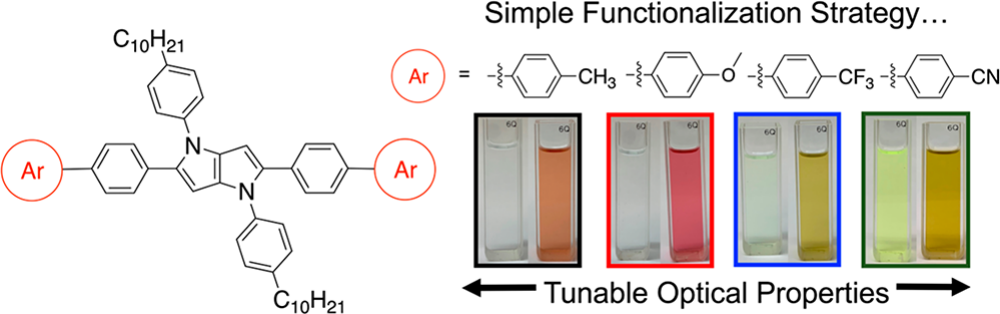

Understanding the influence of peripheral functionality on optoelectronic properties of conjugated materials is an important task for the continued development of chromophores for myriad applications. Here, π-extended 1,4-dihydropyrrolo[3,2-*b*]pyrrole (DHPP) chromophores with varying electron-donating or electron-withdrawing capabilities were synthesized via Suzuki cross-coupling reactions, and the influence of functionality on optoelectronic properties was elucidated. First, chromophores display distinct differences in the UV–vis absorbance spectra measured via UV–vis absorbance spectroscopy in addition to changes in the onset of oxidation measured with cyclic voltammetry and differential pulse voltammetry. Solution oxidation studies found that variations in the electron-donating and -withdrawing capabilities result in different absorbance profiles of the radical cations that correspond to quantifiably different colors. In addition to fundamental insights into the molecular design of DHPP chromophores and their optoelectronic properties, two chromophores display high-contrast electrochromism, which makes them potentially compelling in electronic devices. Overall, this study represents the ability to fine-tune the optoelectronic properties of DHPP chromophores in their neutral and oxidized states and expands the understanding of structure–property relationships that will guide the continued development of DHPP-based materials.

## Introduction

Discrete π-conjugated chromophores are beneficial for studying structure–property relationships compared to polymers due to the ease of introducing subtle structural changes on a molecular level.^1−3^ The control over the structure enables analysis of a single molecular system versus a complex, polydisperse polymer sample. These advantages enable the accurate elucidation of optoelectronic properties based on minimal changes in functionality that otherwise would be convoluted. As such, the thorough understanding of discrete chromophores has made them useful in organic photovoltaic (OPV),^4,5^ organic light-emitting diode (OLED),^6−11^ redox,^12−15^ and bioimaging^16−19^ applications. One molecular scaffold that has emerged as a useful building block for conjugated materials is 1,4-dihydropyrrolo[3,2-*b*]pyrrole (DHPP). Part of the interest in DHPPs is due to their simple synthesis, ease of purification, and expansive design space on the periphery of the molecule through the choice of starting materials. DHPPs are electron-rich scaffolds that have found utility in applications such as photocatalysis^20^ and as active-layer materials in organic resistive memory (ORM) devices,^21^ organic field-effect transistors (OFETs),^22^ and OPVs.^23^ The utility of molecular DHPPs in numerous applications highlights their potential usefulness as advanced optoelectronic materials and emphasizes the need to expand our understanding of structure–property relationships of these chromophores.

While the structural diversity of DHPPs is easily manipulated through the choice of anilines and aldehydes that participate in the Fe-catalyzed reaction used for synthesizing DHPPs, examples of π-extended DHPP systems synthesized via metal-catalyzed cross-coupling reactions are less prevalent. Most of the efforts to create π-expanded DHPPs have involved intramolecular couplings that yield fused aromatic chromophores.^24−30^ As it relates to linear extension of the conjugation, Gryko and co-workers reported a cross-metathesis/borylation procedure to create a π-extended DHPP followed by a Sonogashira coupling reaction to tune the fluorescence into the red region of the electromagnetic spectrum (EMS).^26^ Sonogashira and Suzuki cross-couplings also have been used to synthesize π-extended DHPPs that display two-photon absorbance or that were used in dye-sensitized solar cells (DSSCs).^20,31,32^ Furthermore, Gryko and co-workers used a Sonogashira reaction to produce DHPPs functionalized through the 3,6-positions of the pyrrolopyrrole scaffold,^32^ while work by other groups used direct arylation to obtain DHPP analogs functionalized through the same positions. The chromophores reported by the Gryko and the Vullev groups displayed high fluorescence quantum yields that may make them useful as fluorescent dyes for suitable applications.^33,34^ Still, the scope of coupling partners remains limited, and most reaction yields were modest for intramolecular,^25,26,35^ Sonogashira,^31,32^ Suzuki,^36^ and direct arylation coupling reactions.^34^ While these efforts highlight the ability to functionalize DHPPs through metal-catalyzed reactions, there still is a need to expand the structural diversity of building blocks that efficiently couple with DHPP in these types of reactions or even in high-yielding polymerizations.

The synthetic simplicity, structural tailorabilty, and expansive applicability of DHPPs motivated our group to synthesize and report the first example of a “synthetically simple” DHPP-containing copolymer.^37^ The resulting polymer was solution-processable and displayed yellow-to-black electrochromism with an applied electrochemical potential, which demonstrated that DHPP-based materials may be useful as multicolored electrochromes. However, for DHPPs to realize utility in electrochromic applications, it would be necessary to establish structure–property relationships that relate the choice of comonomers to optical, redox, and color properties of both neutral and oxidized species.

Along these lines, herein, this study reports how altering subtle structural changes of molecular coupling partners manipulates optoelectronic properties of π-extended DHPPs as neutral and oxidized molecules. A family of π-extended DHPPs was first designed based on differences in the electron-donating or -withdrawing capabilities of peripheral substituents and was synthesized using Suzuki cross-coupling reactions. Investigation into the optical properties of the resulting chromophores demonstrates that going from electron-withdrawing to electron-donating substituents, the UV–vis absorbance shifts from the visible to UV region of the EMS, and the onsets of oxidation were lowered, as measured by cyclic voltammetry and differential pulse voltammetry. Upon chemical oxidation, most of the chromophores transition from the UV region of the EMS into the visible region to achieve distinctly different absorbance profiles. Time-dependent density functional theory (TDDFT) calculations were used to confirm trends associated with the absorbance of neutral and oxidized molecules. Through colorimetry analysis, the color profiles of the DHPP chromophores were found to be quantifiably different and demonstrate the ability to control the color of oxidized DHPP systems. Notably, two of the chromophores display high-contrast color changes that make them suitable candidates for anodically coloring electrochromism. In total, results from this study demonstrate how modular manipulations on the periphery of DHPP chromophores influence properties important for redox-active applications, such as electrochromism, and inspire continued investigations into functional DHPP chromophores.

## Materials and Methods

TDDFT calculations using Gaussian 16^38^ and the B3LYP-631G* functional/basis set were performed to elucidate the optical properties of the DHPP molecules that are synthetic targets. First, the molecules were constructed in Gaussview, and geometry optimization was performed to ensure the correct geometry was used in the subsequent calculations. Next, excited-state calculations were run to understand the positioning of the radical cation absorbance. After completion, the data were collected, normalized to the absorbance maximum, and plotted in Origin to report calculated UV–vis absorbance spectra.

Comprehensive details of synthetic protocols and characterization are compiled in the Supporting Information. All materials used in synthetic protocols were purchased from commercial sources and used as received, unless otherwise stated. Anhydrous tetrahydrofuran (THF), dichloromethane (DCM), and toluene were obtained from a Pure Process Technology GC-SPS-7 Glass Contour 800L Solvent Purification System, stored under argon (Ar), and degassed with Ar for 15 min before use. All column chromatography purifications used 60 Å silica gel (200–400 mesh). ^1^H and ^13^C NMR spectra were collected on a Bruker Advance III HD 400 MHz NMR spectrometer with a nominal concentration of 5 mg/mL in CDCl_3_. Peaks are referenced to the residual CHCl_3_ peak (^1^H: δ = 7.26 ppm; ^13^C: δ = 77.23 ppm). Melting point ranges were obtained by depositing samples in borosilicate glass capillary tubes before using a DigiMelt MPA 160 instrument to record the melting temperatures. Optical absorbance spectra of solutions with nominal concentrations of 10–20 mM in toluene or DCM for the molecules were acquired using a Varian Cary 60 Scan single-beam UV–vis–near-IR spectrophotometer scanning from 300 to 800 nm. Solution oxidation experiments involved titrating each solution dropwise with a 0.6 mg/mL Fe(ClO_4_)_3_·*x*H_2_O solution in ethyl acetate until the radical cation peak reached its maximum absorbance intensity. Next to the UV–vis absorbance spectra are photographs of neutral and oxidized solutions in quartz cuvettes after adding the maximum amount of oxidant. Photographs are presented without manipulation, except for cropping. Color coordinates were calculated based on the Commission Internationale de l’Eclairage 1976 L*a*b* color standards using a D50 illuminant as a 2° observer. Cyclic voltammetry (CV) and differential pulse voltammetry (DPV) measurements were performed with a CH Instruments Electrochemical workstation (CHI660D), using a glassy carbon electrode as the working electrode, a Ag/AgCl reference electrode (calibrated versus the Fc/Fc^+^ redox couple, *E*_1/2_ = 46 mV), and a Pt flag as the counter electrode. A 50 mV/s scan rate was used for all electrochemical measurements. An electrolyte solution of 0.5 M tetrabutylammonium hexafluorophosphate (TBAPF_6_, 98%) in anhydrous DCM was used for all electrochemical measurements. The cell and working electrode used to measure the CV and DPV data for one chromophore were an SEC-C thin-layer quartz glass spectroelectrochemical cell with a platinum gauze working electrode. Photography was performed in a light booth designed to exclude outside light with controllable LED lighting above, providing illumination. A Canon Rebel T7 camera with an 18–55 mm lens was used to capture images. Images are presented without manipulation except for cropping. There are no hidden risks or hazards to declare for this work.

## Results and Discussion

**Scheme 1:**
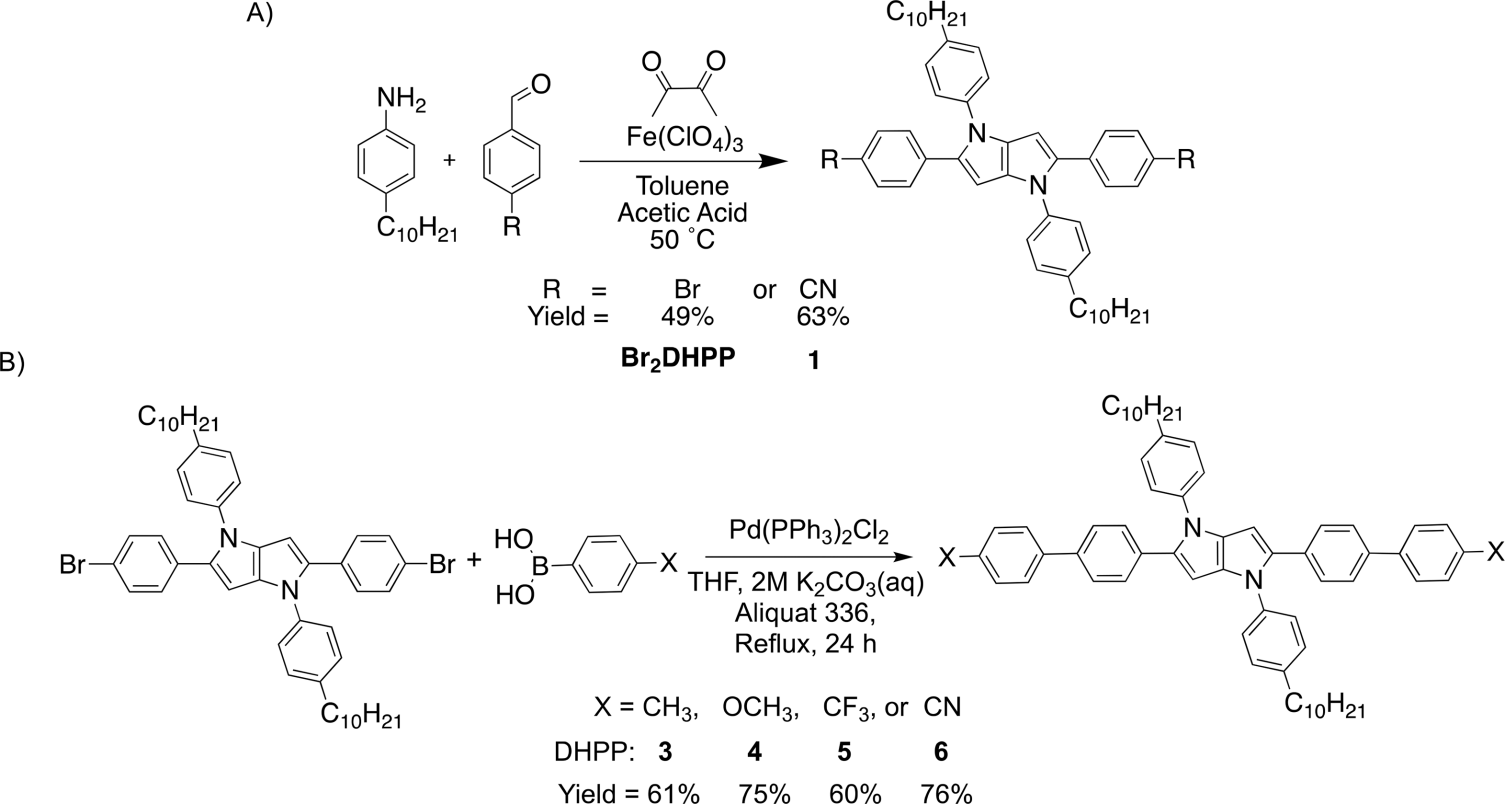
(A) Synthesis of Br_2_DHPP and DHPP **1** via the Fe(III)-Catalyzed Multicomponent Reaction Using Protocols from Gryko and Coworkers.^32^ (B) Synthesis of π-Extended DHPP Chromophores with Various Electronic Character via Suzuki Cross-Coupling Reactions and Their Corresponding Yields

The motivation to study structural effects on the radical cation required the synthesis of DHPP chromophores with various functionalities. Here, we used Suzuki cross-coupling reactions to attain a structurally diverse family of π-extended DHPP chromophores. First, the halogenated starting material Br_2_DHPP was obtained via the Fe-catalyzed multicomponent reaction procedure adopted by our group and was used in Suzuki cross-coupling reactions (Scheme 1A).^37^ The *para*-substituted chromophores, including methyl (DHPP **3**), methoxy (DHPP **4**), trifluoromethyl (DHPP **5**), and cyano (DHPP **6**) substituents, were synthesized in addition to chromophores with substituents at various positions around the benzene ring or alternative aromatics (DHPPs **7**–**12**, Scheme S2). After workup and purification, the π-extended DHPPs were obtained in moderate-to-high yields that were consistent with previous reports involving Pd-catalyzed reactions of DHPPs.^25,26,32,35,39,40^ The structure and purity of the DHPPs were confirmed via ^1^H and ^13^C NMR in tandem with melting point experiments, all of which can be found in the Supporting Information as Figures S1–24 and Table S1. Overall, these efforts represent a robust route to achieving π-extension through the 2,5-positions of DHPP chromophores as an additional strategy for tailoring DHPP dyes.

**Figure 1:**
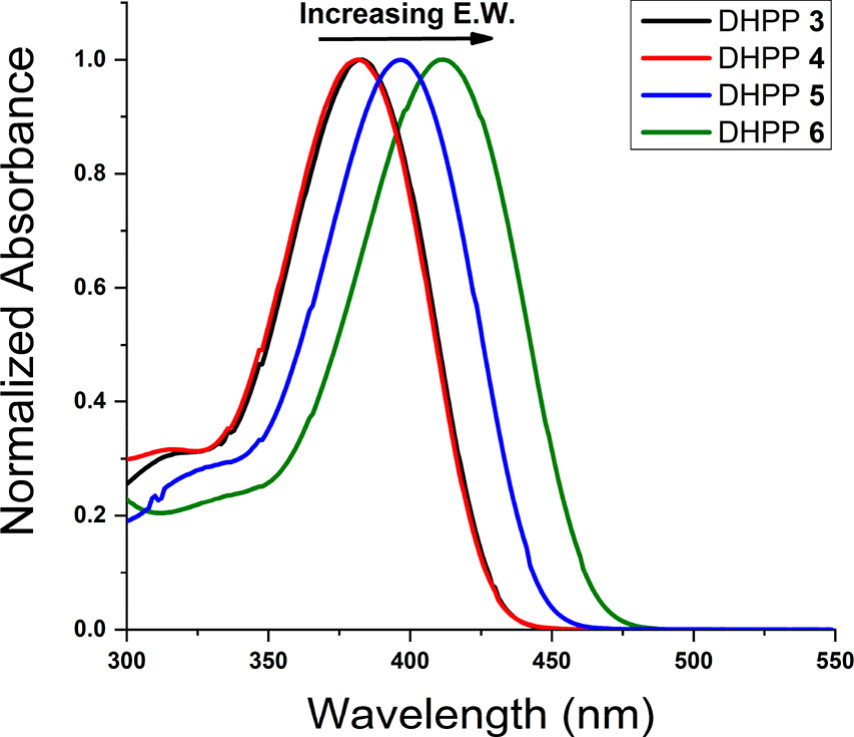
UV–vis absorbance spectra for π-extended DHPP chromophores with various functionalities at the *para-*position of the aromatic coupling partner. Absorbance experiments involved measuring DHPP solutions with nominal concentrations of ∼10 mM in toluene while scanning from 300 to 800 nm.

Once the structure and purity were confirmed, the UV–vis absorbance spectra of all the DHPPs were measured (Figure 1 and Table S2). Specifically, DHPP **3** and **4** had similar absorbance maxima (λ_max_) values at 383 and 382 nm, while DHPP **5** was red-shifted compared to those two DHPPs at 397 nm. DHPP **6** exhibited a further red shift to 412 nm, suggesting that increasing the electron-withdrawing strength results in a decrease in the energy gap between the highest occupied molecular orbital (HOMO) and lowest unoccupied molecular orbital (LUMO) relative to DHPP **5**.^41^ The reduction in the energy gap is attributed to the push–pull effect from the electron-rich DHPP backbone to the electron-deficient pendant.^42,43^ Notably, DHPPs **3**, **4**, and **5** mostly absorbed within the UV region of the EMS, which indicated a diminished push–pull effect with decreasing electron-withdrawing effects and is consistent with the DHPP core being highly electron-rich. Detailed discussion describing the optical properties of DHPPs **7**–**12** is located in the Supporting Information (Figure S25 and Table S2). The UV–vis results are consistent with previously studied π-extended DHPPs where λ_max_ values between ∼368–434 nm were measured and demonstrates the ability to manipulate the absorbance of various DHPP scaffolds.^31,44^ A fundamental investigation of how increasing the π-conjugation of DHPPs, by comparing DHPP **1** and DHPP **6**, is also included in the Supporting Information as Figures S26–28 and Tables S3 and S4. In short, intuitive changes in optoelectronic properties with increasing conjugation, such as red-shifted absorbance and lowered oxidation potentials, were observed when comparing DHPP **1** to DHPP **6**. With the goal of understanding how minimal changes to peripheral substituents influence optoelectronic properties of π-extended DHPPs, further discussion in the text of the paper will focus on *para*-functionalized aromatics with differing electronic effects.

**Figure 2:**
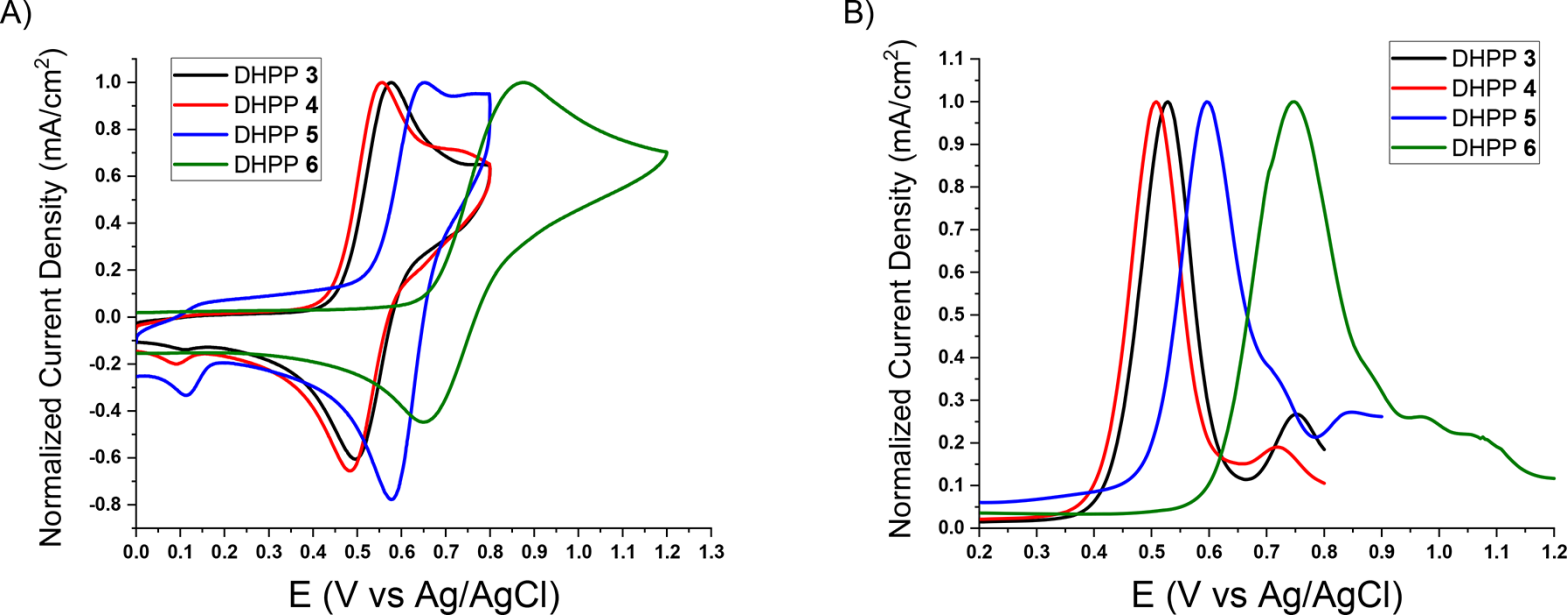
Comparison of the redox response of π-extended DHPPs **3**–**6** via (A) CV and (B) DPV. Measurements were performed using a 0.5 M TBAPF_6_/DCM supporting electrolyte and the Ag/AgCl reference electrode (*E*_1/2_ = 0.46 V vs Fc/Fc^+^).

Turning to substitution effects on the redox response of π-extended DHPPs, cyclic voltammetry (CV) and differential pulse voltammetry (DPV) were used to measure onsets of oxidation and observe the reversibility of *para*-functionalized DHPPs. Fundamentally, the CV and DPV agreed where distinct oxidation peaks were present for the DHPPs (Figure 2). All four DHPPs displayed distinct oxidations and reductions with relatively low onsets of oxidation. Notably, there was not a significant difference between the onset of oxidation of DHPP **3** and DHPP **4** with onset potentials ∼0.41 and ∼0.39 V (vs Ag/AgCl), respectively. These results demonstrate that an electron-neutral and an electron-donating functionalized π-extended coupling partner exhibit the same relative redox activity due to the already electron-rich nature of the DHPP backbone (Figure 2 and Table 1) and that these results agree with the minimal changes measured in UV–vis absorbance experiments. However, when comparing DHPP **5** to DHPP **3** and DHPP **4**, there was an increase in the onset from ∼0.4 to ∼0.51 V (vs Ag/AgCl) due to the electron-withdrawing nature of the −CF_3_ functionality in DHPP **5** (Figure 2 and Table 1). DHPP **6** has the highest onset of oxidation of ∼0.58 V (vs Ag/AgCl) due to the high electronegativity of the cyano group deepening the HOMO energy level.^41^ The similar energy gaps reported in Table 1 (*E*_gaps_ ≈ 2.6–2.8 eV) for each DHPP may be attributed to the disrupted conjugation between the pyrrolopyrrole backbone and the π-extended substituents due to the large pyrrolopyrrole–benzene dihedral angle (∼35°) and the benzene–benzene dihedral angles (∼30°).^37,44,46,47^ Ultimately, these results demonstrate how the choice of coupling partner enables control of the onset of oxidation through alterations in the electronic character without drastically manipulating the optical band gaps.

**Table 1:** Electronic Properties of π-Extended DHPP Chromophores Obtained from Electrochemical and Optical Measurements

Chromophore	*E*_onset_^ox^ (V)	HOMO (eV)^a^	LUMO (eV)^b^	*E*_gap_ (eV)^c^
**3**	0.41	–5.5	–2.7	2.8
**4**	0.39	–5.5	–2.7	2.8
**5**	0.51	–5.6	–3.0	2.6
**6**	0.58	–5.7	–3.1	2.6

a Calculated using HOMO = −(*E*_onset_^ox^ + 5.12 eV).

b Calculated from absorbance onset given eV = 1240/λ_onset_ + HOMO.

c Calculated using *E*_gap_ = (LUMO – HOMO); all equations are adopted from Cardona and co-workers.^45^

**Figure 3:**
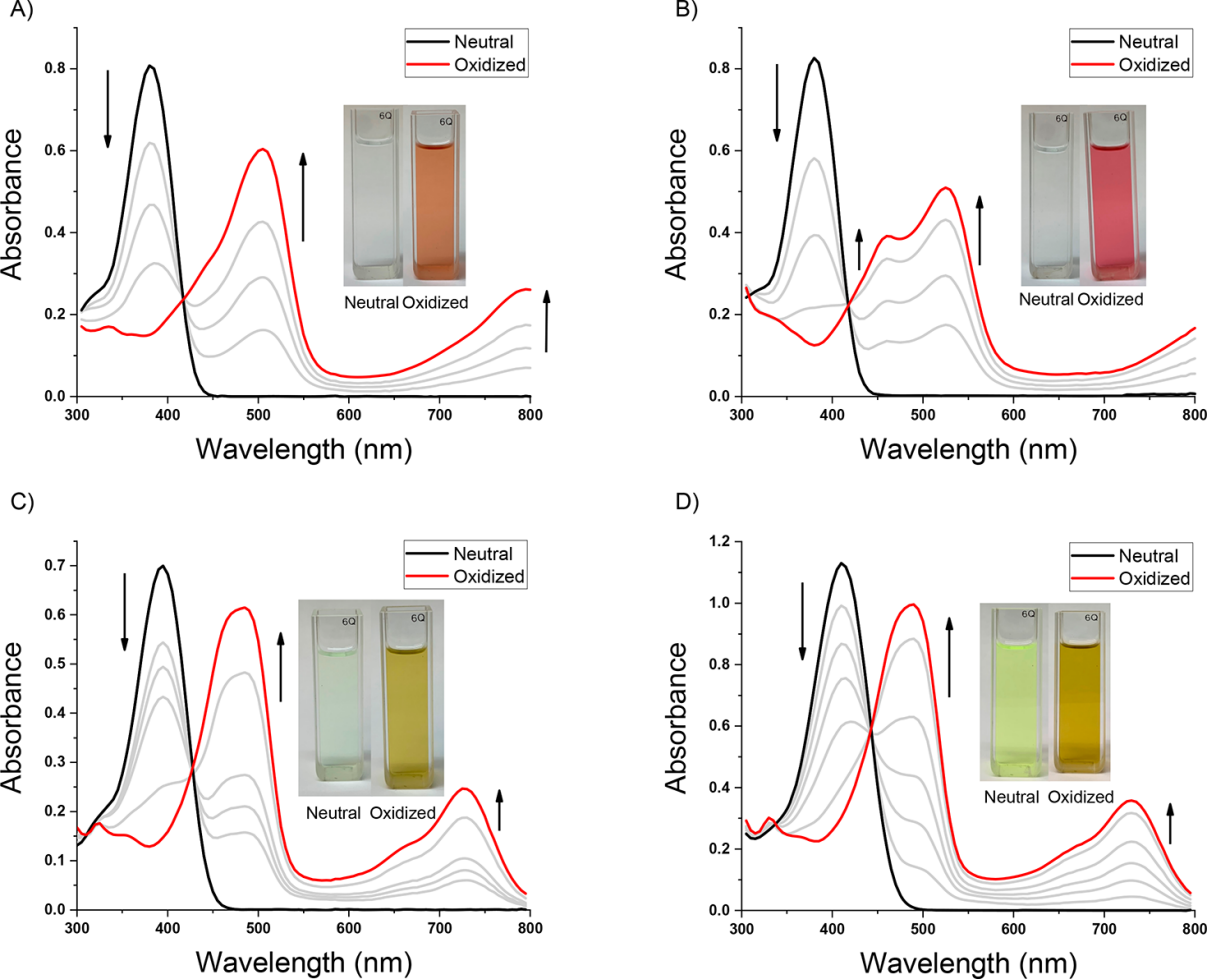
Solution oxidation spectra of (A) DHPP **3**, (B) DHPP **4**, (C) DHPP **5**, and (D) DHPP **6** in DCM using 0.06 mg/mL Fe(ClO_4_)_3_·*x*H_2_O in ethyl acetate as the dopant. These spectra display changes in the UV–vis absorbance spectra with an increasing dopant concentration to elucidate the ability to manipulate the position of the radical cation absorbance.

After understanding the redox activity of the DHPPs, in addition to elucidating substituent influence on optical properties of neutral molecules, efforts shifted to investigate the ability of DHPPs to be chemically oxidized and understanding the optical response of radical cations. Upon oxidation, the changes in absorbance with increasing dopant concentration were monitored with UV–vis absorbance spectroscopy. Solution oxidation experiments were performed for all four *para*-functionalized DHPPs (DHPPs **3**–**6**) by doping the chromophores via titration with a 0.06 mg/mL Fe(ClO_4_)_3_·*x*H_2_O solution to elucidate how the substituent functionality of the coupling partner impacts the absorbance of the radical cation. The transition from neutral to oxidized molecules is shown in Figure 3, and the absorbance maxima of the radical cations are reported in Table 2. Upon chemical doping and formation of the radical cation, DHPPs **3**–**6** shift further into the visible region of the EMS and display characteristics of two absorbance features corresponding to transitions from singly occupied molecular orbitals (SOMOs) to the LUMO, specifically SOMO-α → LUMO-α and SOMO-β → LUMO-β transitions.^48−52^ The high-energy absorption SOMO-α → LUMO-α of the radical cations with absorbance maxima (λ_max_^α^) of DHPP **3** is ∼ 505 nm while DHPP **4** λ_max_^α^ ∼ 525 nm with a distinct shoulder around 460 nm that may be attributed to radical dimerization.^48,53−55^ The red shift of the cation absorbance from DHPP **3** to DHPP **4** is consistent with the electron-donating capabilities of the −OMe group in the chromophore. DHPP **5** and **6** have similar SOMO-α → LUMO-α transitions with DHPP **5** ∼ 485 nm and DHPP **6** ∼ 490 nm. There is a slight blue shift for DHPP **5** compared to DHPP **6** because of the reduced electron-withdrawing effects of the −CF_3_ group of DHPP **5** compared to the −CN group of DHPP **6**. The long-wavelength absorption of the radical cations, or the SOMO-β → LUMO-β, for DHPP **5** and **6** is quite similar in profile and positioning, with an absorbance maximum of the SOMO-β → LUMO-β (λ_max_^β^) ∼ 725 nm for DHPP **5** and ∼ 730 nm for DHPP **6**. Alternatively, the SOMO-β → LUMO-β transitions for DHPP **3** and DHPP **4** are noticeably red-shifted (λ_max_^β^ > 800 nm) compared to DHPP **5** and **6**, consistent with previous work showing that increasing the electron-donating character of peripheral substituents shifts the λ_max_^β^ into the NIR region.^50^ The results of the chemical oxidation studies imply that increasing the electron-donating nature at the *para*-position of peripheral substituents is important for manipulating the SOMO-β → LUMO-β transition. These findings are important for eventual color control as it relates to application within high-contrast electrochromism. The solution oxidation results support optical and electrochemical experiments that show the ability to fine-tune the optoelectronic properties through the choice of functionality on the coupling partner. The overall differences in the absorbance profiles for DHPP **3**–**6** enable an investigation into the color profiles of the neutral and radical species to reveal the effects of substituents on application-inspired properties.

**Table 2:** UV–Vis and Color Coordinate Data for the Selected DHPP Chromophores^a^

		***λ***_***ox***_^***max***^ (nm)		Color Coordinates	
Chromophore	***λ***_neu_^max^ (nm)	Somo-α	Somo-β	Neu. (L*a*b*)	Ox. (L*a*b*)
**3**	383	505	>800	100, −1, 3	86, 24,25
**4**	382	525	>800	100, −1, 3	81, 32, 14
**5**	397	485	725	100, −4, 9	90, 3, 42
**6**	412	490	730	100, −11, 37	83, 10, 52

a The neutral and oxidized λ_max_ values correspond to the SOMO-α → LUMO-α and SOMO-β → LUMO-β, while the neutral and oxidized color coordinates are calculated based on mid-day lighting standards (D50 illuminant as a 2° observer).

**Figure 4:**
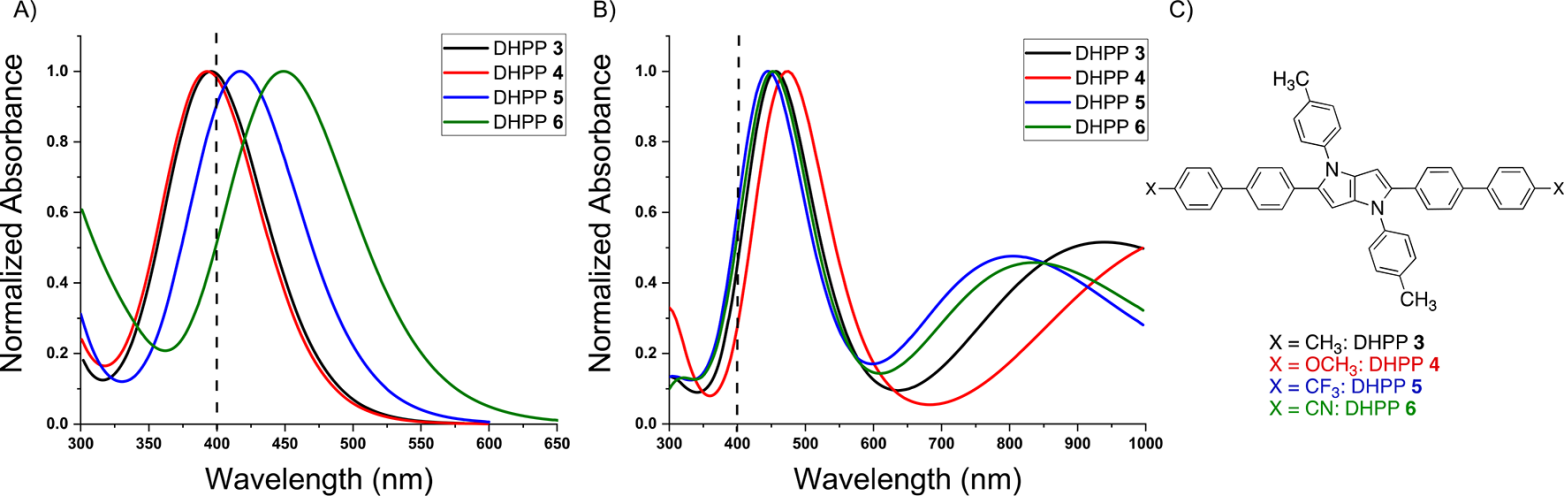
TDDFT UV–vis absorbance spectra for π-extended DHPPs **3**–**6** with varying functionalities as (A) neutral and (B) oxidized chromophores. (C) Representative DHPP structures used for TDDFT calculations. R-groups are truncated to methyl substituents for the sake of simplicity during the calculations.

Four of the π-extended DHPPs, DHPP **3**–**6**, were chosen and modeled using TDDFT to verify the experimental UV–vis results. TDDFT is a powerful tool for elucidating the optical properties of conjugated molecules and has been used to understand and confirm the experimental structure–property relationships of molecular chromophores used in redox-active applications.^56−59^ For this study, the B3LYP-631G* functional and base set was used to confirm the experimental optical properties of the π-extended DHPPs. The calculated neutral UV–vis absorbance spectra align with the experimentally obtained results with only slight variations in the λ_max_ for each DHPP. The trends observed in Figure 1 are confirmed by the TDDFT results within Figure 4A, demonstrating that decreasing the electron-withdrawing nature of the peripheral substituent blue-shifts the absorbance into the UV region of the EMS. Once again motivated to understand optical properties pertinent to electrochromic applications, TDDFT calculations were used to confirm the substituent effects on the absorbance characteristics of radical cations. The calculated spectra support our observations that upon the removal of an electron, the optical transitions will shift to lower energies across the visible spectrum (Figure 4B). The transition to lower energies is in agreement with Figure 3, our previous results,^37^ and observations reported by the Reynolds Group.^48−51^ Overall, TDDFT provides confirmation that the trends seen within our experimental UV–vis absorbance spectra demonstrate a fundamental understanding of the structure–property relationships for this π-extended DHPP family.

**Figure 5:**
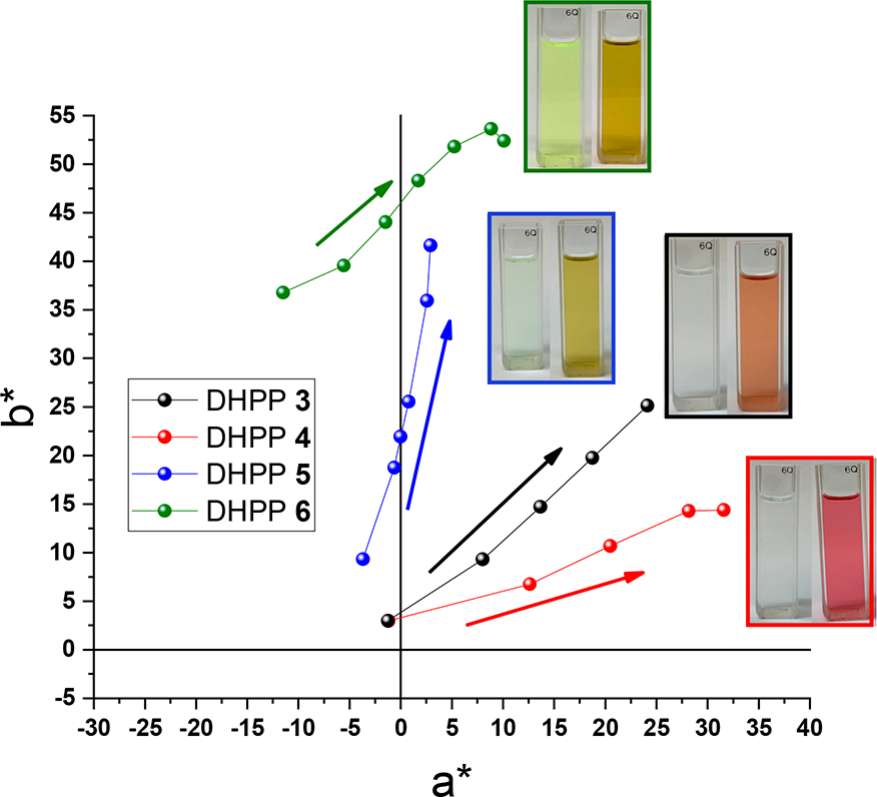
Color coordinates for DHPPs **3**, **4**, **5**, and **6** calculated based on the absorbance change with respect to varying concentrations of chemical dopant. The data illustrate the color control achieved by varying the electronic character through the peripheral substitution of DHPPs. Arrows represent the color track evolving from neutral to oxidized solutions.

The ability to systematically control the absorbance of neutral and oxidized molecules paves the way for aesthetically pleasing, color-changing technologies such as “smart” windows or eyewear with precise color control. With the possibility of these DHPP chromophores being used in electrochromic applications, colorimetry is an important tool for quantifying these color differences.^60^ From the UV–vis solution oxidation studies, color coordinate data can be extrapolated, which enables tracking of the changes in color of the solution based on the evolving absorbance profiles. For this research, the CIE L*a*b* color space was used with a D50 illuminant as a 2° observer.^61^ The neutral colors for DHPP **3** and DHPP **4** were found to be similar to L*a*b* of 100, −1, and 3 (Figure 5 and Table 2). The color coordinates correspond to both molecules being highly transmissive solutions as neutral molecules that transition to colored solutions upon oxidation. Upon the respective color changes, DHPP **3** displayed L*a*b* color coordinates of 86, 24, 25 while DHPP **4** transitions to L*a*b* values of 81, 32, 14. These color tracks correspond to both solutions transitioning from highly transmissive solutions as neutral molecules to red-orange and red solutions, respectively. DHPP **5** also exhibited L*a*b* values consistent with a highly color-neutral solution (100, −4, 9). Upon oxidation, the color shifts to 90, 3, 42 to yield a vibrant yellow color in solution. Unlike DHPP **3**–**5**, DHPP **6** had neutral color coordinates of 100, −11, and 37, which is a light-yellow color when placed on the coordinate diagram and is consistent with the red-shifted UV–vis data reported in Figure 3. Upon oxidation, DHPP **6** displayed a shift in its solution color, resulting in L*a*b* color coordinates of 83, 10, and 52, which corresponded to the yellow-to-golden-yellow color change. All of these results are supported by the photographs presented in Figure 5. Overall, the color data support the notion that the functionality and choice of coupling partner influence the radical cation absorbance of DHPP chromophores. By altering the functionality of the coupling partner from electron-donating to electron-withdrawing, the optical and redox properties are readily manipulated, which now serve as a foundational understanding of structure–property relationships to guide continued development of DHPP-containing materials for electrochromic applications.

**Figure 6:**
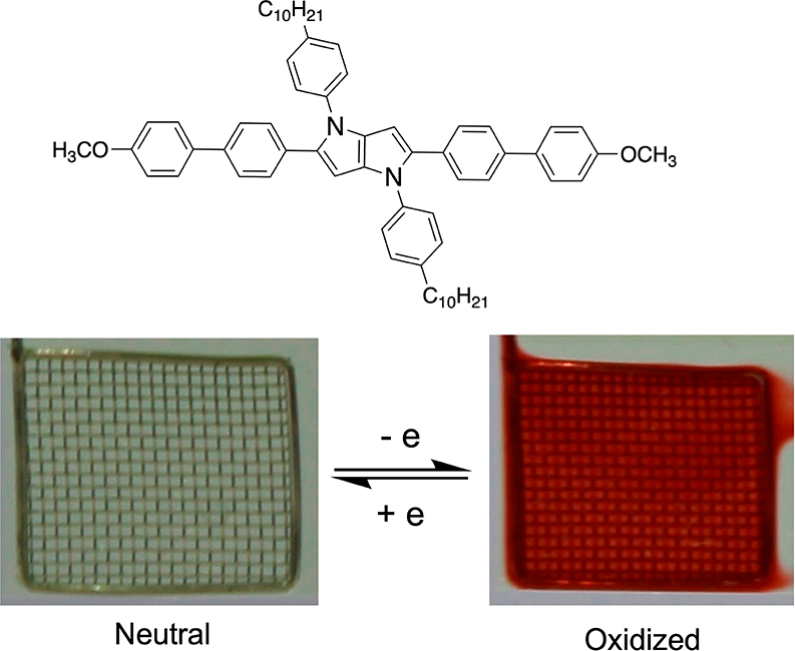
Electrochemical switching experiments using an OTTLE of the color-controlled, high-contrast DHPP chromophore DHPP **4**.

The bulk of this research involved probing how fundamental structural changes influence optoelectronic properties, but the remaining challenge is incorporating these materials into devices. Specifically, while electrochemical and chemical oxidation studies support the notion that these DHPP-based materials will function in electroactive devices, this phenomenon has not been studied in depth. To demonstrate a device proof-of-concept, DHPP **3** and DHPP **4** were dissolved in an electrolyte solution and were electrochemically switched between their neutral and oxidized states. Both DHPPs demonstrated high-contrast electrochemical switching from a relatively transmissive neutral state to either orange or red oxidized states (Figures 6 and S29). Both of these color transitions are consistent with the solution oxidation studies and the colorimetry data. In summary, these results motivate continued investigation into DHPP chromophores as high-contrast electrochromic materials for applications within organic electronic devices.

## Conclusions

Electron-rich pyrrolopyrroles are an emerging class of materials that are garnering attention in numerous optoelectronic applications. For DHPPs to continue to garner attention in various applications, a thorough understanding of how functionalities influence application-inspired properties is concurrently needed. With these considerations in mind, a family of π-extended DHPP chromophores was hypothesized to lend insight into the effects of structural variations, specifically changes in the electron-donating and electron-withdrawing character, on optical properties of neutral and oxidized chromophores. Chromophores are accessed through robust, high-yielding Pd-catalyzed Suzuki cross-coupling reactions between a dibrominated DHPP and the corresponding boronic acid coupling partner. The resulting optical properties of neutral and oxidized chromophores were studied via UV–vis absorbance spectroscopy and reveal a dependence between the substitution patterns and functionality on the absorbance characteristics. Specifically, as the electron-donating nature of the peripheral substituent is increased, there is an observed blue-shift in the absorbance of neutral molecules and a red-shift in spectra measured for chemically oxidized chromophores. These results were further confirmed via TDDFT, and the agreement between theory and experiment opens the opportunity for theory-guided DHPP-containing material. Electrochemical studies also confirm substituent effects influence redox properties and that the π-extended DHPPs possess relatively low oxidation potentials (∼0.4 – 0.6 V vs Ag/AgCl). The low onsets of oxidation may render them useful in redox-active applications and motivated the study of chromophores as electrochromes. Notably, two π-extended DHPPs display transmissive-to-colored transitions upon oxidation and demonstrate the potential utility of DHPPs as high-contrast anodically coloring electrochromes. Combined, these results provide strategies for tuning the optoelectronic properties of DHPP molecules and expand their utility as materials used in electrochemical applications. The large number of verified functionalization strategies of DHPPs and their ability to participate in cross-coupling reactions suggest DHPP chromophores may find applicability in optoelectronic applications such as high-contrast electrochromism as molecules or polymers.

## Supporting Information Available

Materials and methods, computational details, synthetic procedures, ^1^H and ^13^C of molecules, and other ancillary data and discussion (PDF)
