# Supplementary material for: Relating Design and Optoelectronic Properties of 1,4-Dihydropyrrolo[3,2-b]pyrroles Bearing Biphenyl Substituents
Source: J Phys Chem B. 2023 Aug 10;127(33):7352–60. doi: 10.1021/acs.jpcb.3c03061 (PMC10461294; doi:10.1021/acs.jpcb.3c03061)
Supplement: Supplementary file 1 — jp3c03061_si_001.pdf [file jp3c03061_si_001.pdf]

Supporting Information

Relating Design and Optoelectronic Properties of 1,4-Dihydropyrrolo[3,2-*b*]pyrroles Bearing Biphenyl Substituents

Allison M. Hawks, Drake Altman, Ryan Faddis, Ethan Wagner, Kenneth-John J. Bell, Ariane Charland-Martin, and Graham S. Collier*

Department of Chemistry and Biochemistry, Kennesaw State University, Kennesaw GA, 30144, USA

Synthesis

Synthesis of Functionalized DHPP Molecules via an Fe(III)-Catalyzed Multicomponent Reaction

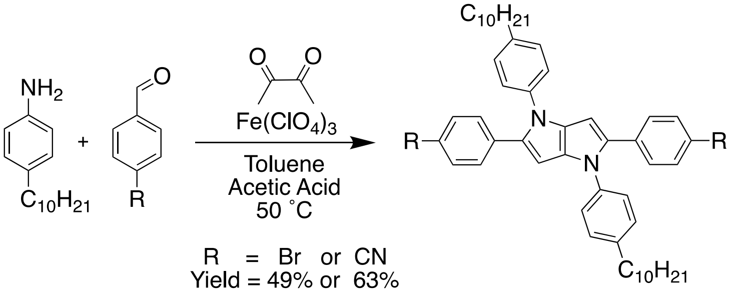

Scheme S1. General synthesis of the brominated DHPP monomer (Br_2DHPP) and DHPP **1** using protocols adopted from our previous work.[1]

The synthetic protocol for DHPPs was adopted from previous work reported by our group.[1] Briefly, *n*-decylaniline (8 mmol) and *p*-bromobenzaldehyde (8 mmol) or 4-cyanobenzaldehyde (8 mmol) are added to a solution of toluene (6 mL) and glacial acetic acid (6 mL) inside a 25 mL round bottom flask equipped with a magnetic stir bar. The reaction mixture was stirred for 1 h in an oil bath set to 50 ˚C. Once the initial heating time was completed, $Fe(ClO_4)_3$□xH_2O (0.085 g)

was added to the reaction flask, followed by 2,3-butanedione (0.35 mL, 4.00 mmol). After these additions, the reaction was allowed to stir at 50 ˚C overnight. The next day the reaction was removed from heat and allowed to cool to room temperature. The reaction precipitate was collected via vacuum filtration and washed with cold MeOH and acetone until a white or pale-yellow solid remained on the filter paper. The precipitate was transferred to a vial and dried overnight under vacuum. After structural analysis, each molecule/monomer was confirmed to be the desired product.

2,5-bis(4-bromophenyl)-1,4-bis(4-*n*-decylphenyl)-1,4-dihydropyrrolo[3,2-*b*]pyrrole (Br_2DHPP): Analysis of the analytical data supports successful synthesis of the desired product and agrees with prior reports.[1]

2,5-bis(4-cyanophenyl)-1,4-bis(4-*n*-decylphenyl)-1,4-dihydropyrrolo[3,2-*b*]pyrrole (1): Yellow Solid. Yield: 1.88 g (63%) ^{1}H NMR (400 MHz, $CDCl_3$), δ: 0.91 (t, 6H), 1.31-1.37 (m, 29H), 1.64-1.69 (m, 4H), 2.38 (s, 1H), 2.67 (t, 4H), 6.49 (s, 2H), 7.17-7.26 (m, 10H), 7.30 (d, 6H), 7.49 (d, J = 8.5 Hz, 4H). The ^{1}H NMR spectrum agrees with prior reports.[2,3]

General Suzuki Cross-Coupling Procedure for π-Extended DHPPs

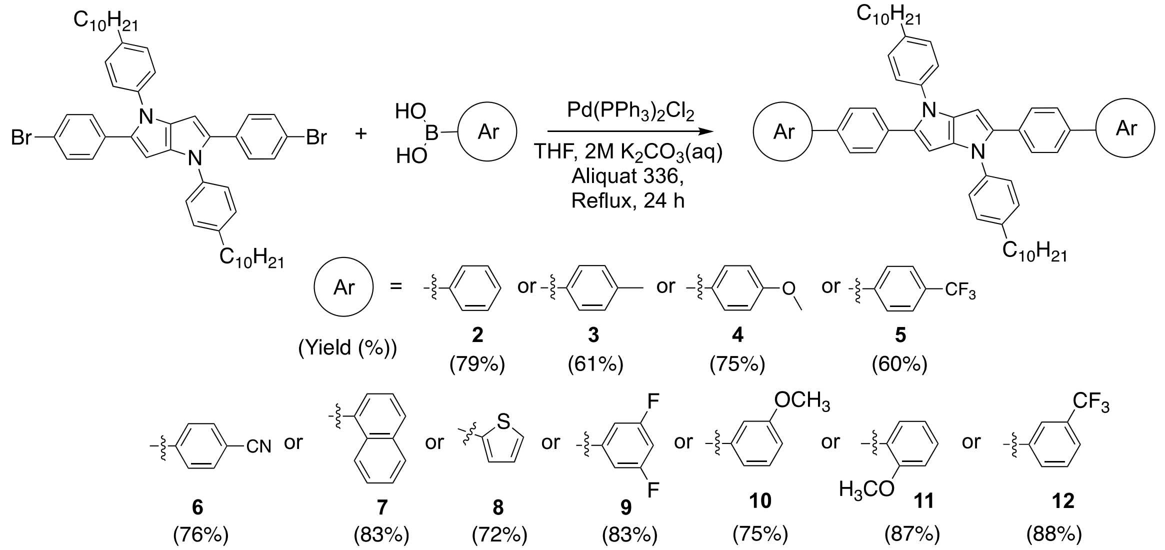

Scheme S2. General Suzuki cross-coupling reaction for π-extended DHPP chromophores.

In a 10 mL round bottom flask equipped with a Teflon stir bar, DHPP (100 mg, 0.12 mmol), 2.2 molar equiv. of the corresponding aryl-boronic acid (0.264 mmol), 2 mol% of $Pd(PPh_3)_2Cl_2$, and one drop of Aliquat 336 were combined. A reflux condenser was attached along with a rubber septum, and the reaction flask was rendered inert via vacuum/refill cycles (3×) with Argon (Ar). Subsequently, 2 mL of degassed THF and 2M K_2CO_3(aq) were added via syringe, and the flask was placed in an oil bath set to 70 °C. The reaction was allowed to stir for at least 20 h before being removed from the oil bath and cooled to r.t.. The crude reaction mixture was added to a separatory funnel, and the flask was rinsed with DCM. The mixture was washed with H_2O (3×20 mL) and extracted with DCM. The organic layers were dried with Na_2SO_4, filtered, and concentrated via rotary evaporation. The product was then purified via column chromatography using a mixture of Hex:DCM as the eluent. Specific mobile phases are listed with the corresponding molecules. The expected product was determined via TLC and pure fractions were collected and concentrated via rotary evaporation. The solid product was then transferred to a vial and dried on the vacuum overnight. The expected products were then confirmed via NMR and elemental analysis.

2,5-bis(4-phenyl)-2,5-bis(phenyl)-1,4-bis(4-*n*-decylphenyl)-1,4-dihydropyrrolo[3,2-*b*]pyrrole (2): Yellow Solid. Yield: 68.9 mg (79%) Mobile phase 3:1 (Hex:DCM). Melting point: 172.1 – 174.5 ˚C ^{1}H NMR (400 MHz, $CDCl_3$), δ: 0.91 (t, 8H), 1.30-1.36 (m, 35H), 1.66-1.69 (m, 5H), 2.66 (t, 4H), 6.49 (s, 2H), 7.22 (d, J = 8.3 Hz, 4H), 7.28 (d, 6H), 7.34 (t, 6H), 7.44 (t, 4H), 7.50 (d, J = 8.2 Hz, 4H), 7.62 (d, J = 7.5 Hz, 4H). ^{13}C NMR: (400 MHz, $CDCl_3$), δ: 14.1, 22.7, 29.5, 31.4, 31.9, 35.5, 94.6, 125.2, 126.75, 127.1, 128.3, 128.8, 129.1, 132.0, 132.8, 135.5, 137.7, 138.5, 140.5, 140.7. Anal. calc'd for $C_{62}H_{70}N_2$: C 88.31; H 8.37; N 3.32 Found: C 87.21; H 8.55; N 3.22.

2,5-bis(4-toyl)-2,5-bis(phenyl)-1,4-bis(4-*n*-decylphenyl)-1,4-dihydropyrrolo[3,2-*b*]pyrrole (3): Yellow Solid. Yield: 66.5 (64%) Mobile phase 3:1 (Hex:DCM). Melting point: 169.0 ˚C ^{1}H NMR (400 MHz, $CDCl_3$), δ: 0.91 (t, 7H), 1.30-1.36 (m, 32H), 1.65-1.69 (m, 4H), 2.41 (s, 6H), 2.66 (t, 4H), 6.47 (s, 2H), 7.19-7.31 (m, 22H), 7.46-7.52 (m, 9H). ^{13}C NMR: (400 MHz, $CDCl_3$), δ: 14.1, 21.1, 22.7, 29.4, 29.5, 29.7, 31.4, 31.9, 35.5, 94.5, 125.2, 126.5, 126.6, 128.3, 129.0, 129.5, 131.9, 132.5, 135.6, 136.9, 137.8, 138.4, 140.5. Anal. calc'd for $C_{64}H_{74}N_2$: C 88.22; H 8.56; N 3.22 Found: C 88.06; H 8.65; H 3.23.

2,5-bis(4-methoxyphenyl)-2,5-bis(phenyl)-1,4-bis(4-*n*-decylphenyl)-1,4-dihydropyrrolo[3,2-*b*]pyrrole (4): Yellow Solid. Yield: 80.8 mg (75%) Mobile phase 2:1 (Hex:DCM). Melting point: 187.0 – 189.2 ˚C ^{1}H NMR (400 MHz, $CDCl_3$), δ: 0.90 (t, 7H), 1.30-1.36 (m, 33H), 1.65-1.69 (m, 5H), 2.65 (t, 4H), 3.87 (s, 7H), 6.46 (s, 2H), 6.98 (d, J = 8.8 Hz, 4H), 7.20 (d, J = 8.4 Hz, 4H), 7.28 (t, 15H), 7.44 (d, J = 8.4 Hz, 4H), 7.54 (d, J = 8.8 Hz, 4H). ^{13}C NMR: (400 MHz, $CDCl_3$), δ: 14.1, 22.7, 29.4, 29.5, 29.7, 31.4, 31.9, 35.5, 55.4, 94.5, 114.2, 125.2, 126.2, 127.8, 128.3, 129.0, 131.9,132.2, 133.3, 135.6, 137.8, 138.1, 140.5, 159.1. Anal. calc'd for $C_{64}H_{74}N_2O_2$: C 85.10; H 8.26; N 3.10 Found: C 85.19; H 8.33; N 3.17.

2,5-bis(4-trifluoromethylphenyl)-2,5-bis(phenyl)-1,4-bis(4-*n*-decylphenyl)-1,4-dihydropyrrolo[3,2-*b*]pyrrole (5): Yellow Solid. Yield: 69.5 mg (59%) Mobile phase 5:1 (Hex:DCM). Melting point: 190.0 – 192.0 ˚C ^{1}H NMR (400 MHz, $CDCl_3$), δ: 0.90 (t, 7H), 1.29-1.36 (m, 31H), 1.64-1.69 (m, 4H), 2.67 (t, 4H), 6.49 (s. 2H), 7.22 (d, J = 8.5 Hz, 4H), 7.27 (d, 12H), 7.35 (d, J = 8.4 Hz, 4H), 7.50 (d, J = 8.4 Hz, 4H), 7.67-7.72 (m, 8H). ^{13}C NMR: (400 MHz, $CDCl_3$), δ: 14.1, 22.7, 29.4, 29.5, 29.6, 31.4, 31.9, 35.5, 94.8, 125.2, 125.67-125.7, 126.9-127.0, 128.4, 129.1, 132.3, 133.7, 135.4, 136.9, 137.6, 140.8, 144.2. Anal. calc'd for $C_{64}H_{68}F_6N_2$: C 78.50; H 7.00; F 11.64; N 2.86 Found: C 78.07; H 7.15; N 2.85.

2,5-bis(4-cyanophenyl)-2,5-bis(phenyl)-1,4-bis(4-*n*-decylphenyl)-1,4-dihydropyrrolo[3,2-*b*]pyrrole (6): Vibrant yellow solid. Yield: 81.2 mg (76%) Mobile phase 1:1 (Hex:DCM). Melting point: 222.6 – 225.4 ˚C ^{1}H NMR (400 MHz, $CDCl_3$), δ: 0.90 (t, 7H), 1.29-1.40 (m, 33H), 1.64-1.69 (m, 5H), 2.66 (t, 4H), 6.50 (s, 2H), 7.21-7.28 (m, 19H), 7.35 (d, J = 8.4Hz, 5H), 7.49 (d, J = 8.5 Hz, 4H), 7.69-7.73 (m, 9H). ^{13}C NMR: (400 MHz, $CDCl_3$), δ: 14.1, 22.7, 29.4, 29.5, 29.6, 31.4, 31.9, 35.5, 94.9, 110.6, 119.0, 125.2, 126.9, 127.3, 128.4, 129.2, 132.5, 132.6, 134.1, 135.4, 136.3, 137.5, 140.9, 145.1. Anal. calc'd for $C_{64}H_{68}N_4$: C 86.05; H 7.67; N 6.27 Found: C 85.79; H 7.86; N 6.09.

2,5-bis(4-napthelene)-2,5-bis(phenyl)-1,4-bis(4-*n*-decylphenyl)-1,4-dihydropyrrolo[3,2-*b*]pyrrole (7): Yellow Solid. Yield: 94 mg (83%) Mobile phase 4:1 (Hex:DCM). Melting point: 138.2 – 140.7˚C ^{1}H NMR (400 MHz, $CDCl_3$), δ: 0.89 (t, 6H), 1.27-1.36 (m, 31H), 1.67-1.70 (m, 4H), 2.68 (t, 4H), 6.55 (s, 2H), 7.25 (d, J = 8.3 Hz, 4H), 7.34 (d, J = 8.4 Hz, 4H), 7.39 (s, 8H), 7.45-7.56 (m, 9H), 7.87 (d, J = 8.1 Hz, 2H), 7.92 (d, J = 7.4 Hz, 2H), 7.99 (d, J = 8.3 Hz, 2H). ^{13}C NMR: (400 MHz, $CDCl_3$), δ: 14.1, 22.7, 29.3-29.4, 29.5, 29.6, 31.4, 31.9, 35.5, 94.8, 125.2, 125.4, 125.7, 125.97-126.04, 126.9, 127.6, 128.0, 128.3, 129.1, 129.9, 131.6, 132.0, 132.8, 133.9, 135.6, 137.8, 138.3, 140.0, 140.6. Anal. calc'd for $C_{70}H_{74}N_2$: C 89.12; H 7.91; N 2.97 Found: C 84.85; H 7.86; N 2.83.

2,5-bis(4-thiophene)-2,5-bis(phenyl)-1,4-bis(4-*n*-decylphenyl)-1,4-dihydropyrrolo[3,2-*b*]pyrrole (8): Yellow Solid. Yield: 73.6 mg (72%) Mobile phase 4:1 (Hex:DCM). Melting point: 187.8 – 189.4 ˚C ^{1}H NMR (400 MHz, $CDCl_3$), δ: 0.91 (t, 7H), 1.30-1.36 (m, 31H), 1.64-1.69 (m, 4H), 2.66 (t, 4H), 6.45 (s, 2H), 7.07-7.09 (m, 2H), 7.20-7.30 (m, 21H), 7.49 (d, J = 8.4 Hz, 4H). ^{13}C NMR: (400 MHz, $CDCl_3$), δ: 14.1, 22.7, 29.4, 29.5, 29.7, 31.4, 31.9, 35.5, 94.5, 122.7, 125.2,

125.5, 128.0, 128.3, 129.1, 131.9, 132.1, 132.9, 135.5, 137.7, 140.6. Anal. calc'd for $C_{58}H_{66}N_2S_2$: C 81.45; H 7.78; N 3.28; S 7.50 Found: C 81.56; H 7.86; N 3.21; S 7.69.

2,5-bis(3,5-fluorophenyl)-2,5-bis(phenyl)-1,4-bis(4-*n*-decylphenyl)-1,4-dihydropyrrolo[3,2-*b*]pyrrole (9): Yellow Solid. Yield: 89.3 mg (83%) Mobile phase 5:1 (Hex:DCM). Melting point: 156.9-159.4 ˚C ^{1}H NMR (400 MHz, $CDCl_3$), δ: 0.87 (t, 7H), 1.27-1.33 (m, 31H), 1.61-1.67 (m, 4H), 2.64 (t, 4H), 6.46 (s, 2H), 6.72-6.77 (m, 2H), 7.06-7.11 (m, 4H), 7.18-7.25 (m, 10H), 7.29 (d, *J* = 8.4 Hz, 4H), 7.41 (d, *J* = 8.4 Hz, 4H). ^{13}C NMR: (400 MHz, $CDCl_3$), δ: 14.1, 22.7, 29.3-29.4, 29.5, 29.6, 31.4, 31.9, 35.5, 94.8, 102.2 (d, *J* = 25.5 Hz), 109.4 (t, *J* = 7.0 Hz), 109.6 (d, J = 7.0 Hz), 125.2, 126.6, 128.4, 129.2, 132.4, 133.9, 135.4, 136.1, 137.6, 140.8, 144.0, 162.1 (d, *J* = 13.3 Hz), 164.6 (d, *J* = 13.1 Hz), 164.7-162.2 (d, *J* = 248.5 Hz), 164.5-162.1 (d, *J* = 249.5 Hz). Anal. calc'd for $C_{62}H_{66}F_4N_2$: C 81.37; H 7.27; N 3.06 Found: C 81.07; H 7.34; N 3.05.

2,5-bis(3-trifluoromethylphenyl)-2,5-bis(phenyl)-1,4-bis(4-*n*-decylphenyl)-1,4-dihydropyrrolo[3,2-*b*]pyrrole (10): Yellow Solid. Yield: 101.1 mg (88%) Mobile phase 5:1 (Hex:DCM). Melting point: 135.2 – 141.1 ˚C ^{1}H NMR: (400 MHz, $CDCl_3$), δ: 0.91 (t, 7H), 1.30-1.37 (m, 31H), 1.65-1.72 (m 4H), 2.67 (t, 4H), 6.50 (s, 2H), 7.22-7.29 (m, 9H), 7.35 (d, *J* = 8.4 Hz, 4H), 7.50 (d, *J* = 8.4 Hz, 4H), 7.53-7.61 (m, 4H), 7.78 (d, *J* = 7.5 Hz, 2H), 7.85 (s, 2H). ^{13}C NMR: (400 MHz, $CDCl_3$), δ: 14.1, 22.7, 29.35-29.39, 29.5, 29.6, 31.4, 31.9, 35.5, 94.8, 122.9, 123.56-123.60, 123.7, 125.2, 125.6, 126.8, 128.4, 129.1-129.2, 130.0, 131.0, 131.3, 132.3, 133.6, 135.4, 136.9, 137.6, 140.8, 141.5. Anal. calc'd for $C_{64}H_{68}F_6N_2$: C 78.50; H 7.00; N 2.86 Found: C 78.24; H 7.14; N 2.85.

2,5-bis(3-methoxyphenyl)-2,5-bis(phenyl)-1,4-bis(4-*n*-decylphenyl)-1,4-dihydropyrrolo[3,2-*b*]pyrrole (11): Yellow Solid. Yield: 77.2 mg (75%) Mobile phase 2:1 (Hex:DCM). Melting point: 121.7 – 123.8 ˚C ^{1}H NMR (400 MHz, $CDCl_3$), δ: 0.89-0.92 (m, 9H), 1.30-1.36 (m, 37H), 1.66-

1.69 (m, 5H), 2.66 (t, 4H), 3.88-3.90 (m, 7H), 6.48 (s, 2H), 6.88-6.91 (m, 2H), 7.15 (t, 2H), 7.19-7.22 (m, 7H), 7.26-7.38 (m, 13H), 7.48-7.50 (m, 4H). ^{13}C NMR: (400 MHz, $CDCl_3$), δ: 11.2, 14.1, 22.7, 29.35-29.38, 29.5, 29.6, 31.4, 31.9, 35.5, 55.3, 94.6, 112.6, 119.4, 125.2, 126.8, 128.3, 129.1, 129.7, 132.1, 133.0, 135.5, 137.7, 138.3, 140.6, 142.3, 159.9. Anal. calc'd for $C_{64}H_{74}N_2O_2$: C 85.10; H 8.26; N 3.10 Found: C 84.23; H 8.30; N 3.06.

2,5-bis(2-methoxyphenyl)-2,5-bis(phenyl)-1,4-bis(4-*n*-decylphenyl)-1,4-dihydropyrrolo[3,2-*b*]pyrrole (12): Yellow Solid. Yield: 90.1 mg (87%) Mobile phase 2:1 (Hex:DCM). Melting point: 118.7 – 120.9 ˚C ^{1}H NMR (400 MHz, $CDCl_3$), δ: 0.91 (t, 7H), 1.30-1.36 (m, 31H), 1.66-1.69 (m, 4H), 2.66 (t, 4H), 3.84 (s, 6H), 6.47, (s, 2H), 6.99-7.06 (m, 4H), 7.21 (d, *J* = 8.4 Hz, 4H), 7.28-7.32 (m, 11H), 7.34-7.37 (m, 3H), 7.45 (d, *J* = 8.4 Hz, 4H). ^{13}C NMR: (400 MHz, $CDCl_3$), δ: 11.2, 14.1, 22.7, 29.35-29.38, 29.5, 29.6, 31.4, 31.9, 35.5, 55.3, 94.6, 112.5-112.6, 119.4, 125.2, 126.8, 128.3, 129.1, 129.7, 132.1, 133.0, 135.5, 137.7, 138.3, 140.6, 142.3, 159.9. Anal. calc'd for $C_{64}H_{74}N_2O_2$: C 85.10; H 8.26; N 3.10 Found: C 85.04; H 8.41; N 3.11.

The yields for the *para*-substituted chromophores were adequate at 60-76% which is consistent with previous reports.[4,5] DHPP **7** and **8** were successfully synthesized with adequate yields of 83% and 72%, respectively. The naphthalene yield was still high consistent with previous naphthalene derivatives, demonstrating that there is not significant steric constraints for this synthesis.[5] As a way to investigate the influence of the π-extension on the structure-property relationships of DHPPs, DHPP **1** also was synthesized to compare with DHPP **6**.

Purification of each DHPP chromophore was accomplished via column chromatography and structure and purity were confirmed with ^{1}H and ^{13}C NMR. Figure S1 is the NMR spectra for DHPP **1** where the diagnostic pyrrolopyrrole peak at ~ 6.5 ppm is present and all other protons match previous reports.[2,3] Figure S2 shows the NMR spectra for the starting material Br_2DHPP

and all the protons align with previous reports as well.[1] The ^{1}H and ^{13}C NMR spectra for the π-extended DHPPs also can be found in the SI as Figures S3-S24. All expected protons and carbons were accounted for in the NMR spectra. Importantly, the diagnostic DHPP peak at ~ 6.5 ppm was retained for the π-extended DHPPs, which indicates there was no observation of side products as a result of the pyrrolopyrrole protons being active in direct arylation reactions. Purity was also determined using elemental analysis (EA) where theoretical and calculated values were found to be statistically similar and further validating the purity of the molecules. Melting points were used as another analytical technique for determining purity[6] and all molecules were found to have narrow melting point ranges attributed to a high level of purity. (Table S1).[7] Additionally, each of the synthesized DHPPs have melting points similar to previously published molecular DHPPs (~ 130 ˚C < MP < ~ 220 ˚C).[8] One notable observations arises from variations in the electronic character and the positioning of the substituents altering the strength and stability of the intermolecular forces that influence the melting point. For example, molecules substituted *ortho* or *meta* to the DHPP core have melting points lower than those substituted at the *para* position. Additionally, chromophores substituted with electron-withdrawing substituents have higher melting temperatures. Overall, the three analytical techniques confirm that the molecules are the desired products at a high level of purity which will enable an accurate elucidation of their structure-property relationships.

Table S1. Melting point ranges for DHPPs **2-12**. The narrow temperature ranges are consistent with a high level of purity.

**DHPP**	**2**	**3**	**4**	**5**	**6**	**7**	**8**	**9**	**10**	**11**	**12**
**Melting Point (˚C)**	172-174	169	187-189	190-192	223-225	138-141	188-189	157-159	135-141	122-124	119-121

NMR Characterization

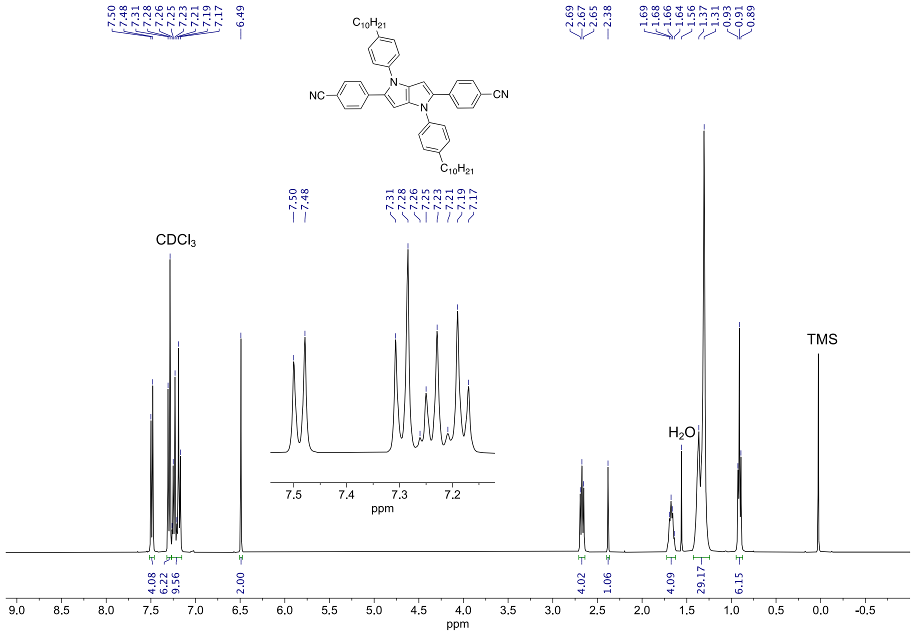

Figure S1. ^{1}H NMR (400 MHz, 25 ˚C, $CDCl_3$) of **1**.

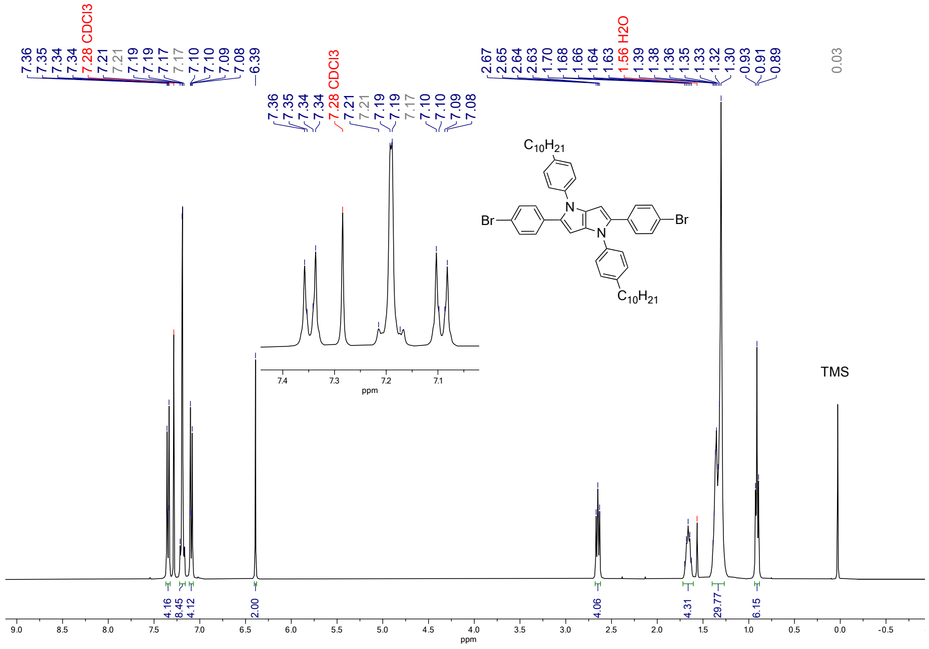

Figure S2. ^{1}H NMR (400 MHz, 25 ˚C, $CDCl_3$) of Br_2DHPP.

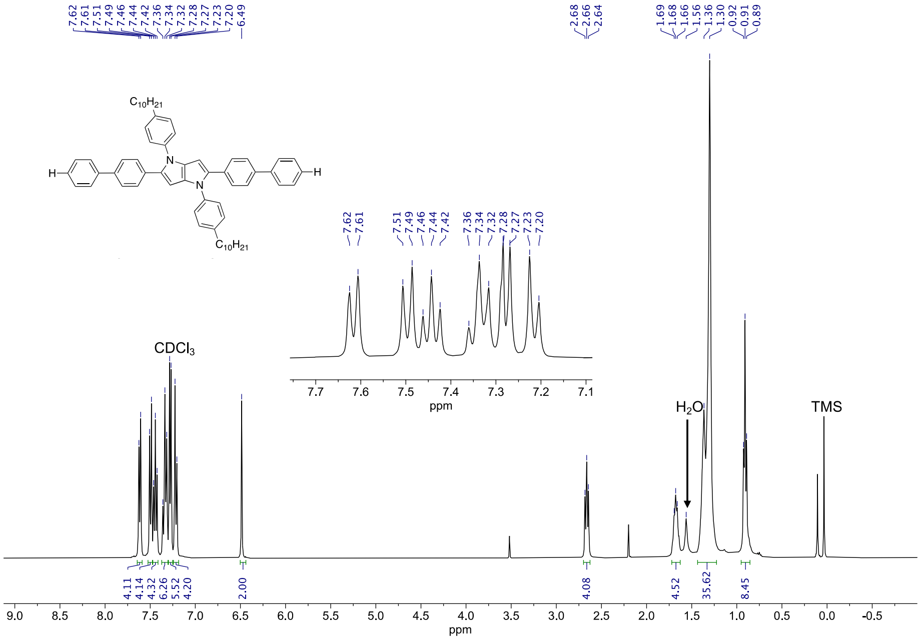

Figure S3. ^{1}H NMR (400 MHz, 25 ˚C, CDCl$_3$) of **2**.

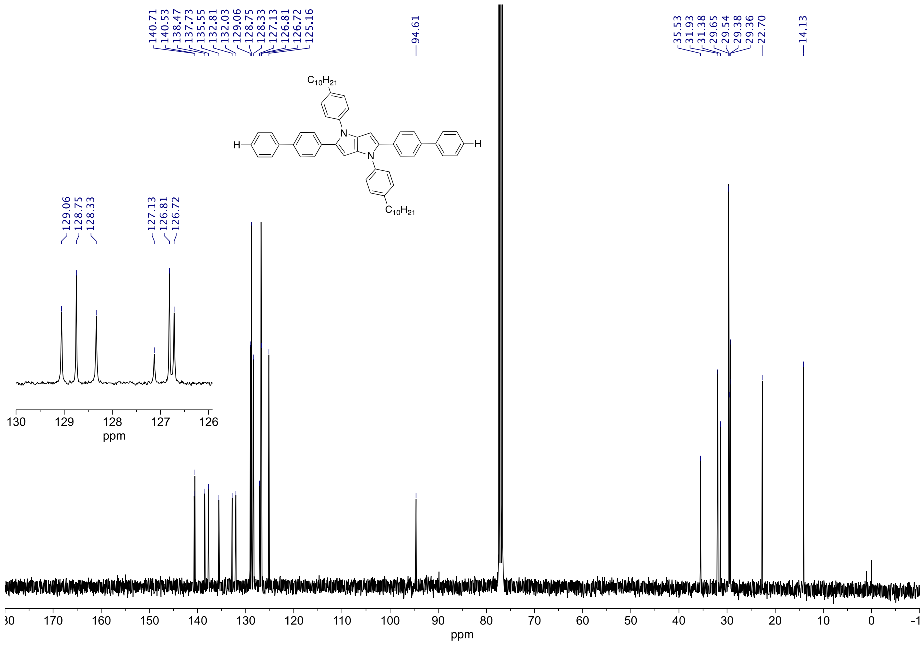

Figure S4. ^{13}C NMR (400 MHz, 25 ˚C, CDCl$_3$) of **2**.

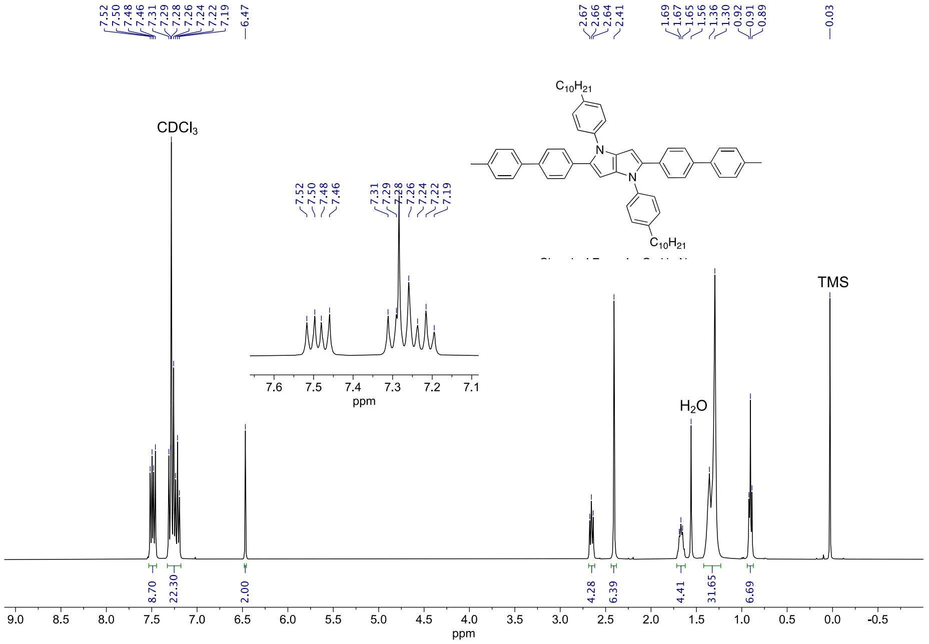

Figure S5. ^{1}H NMR (400 MHz, 25 ˚C, $CDCl_3$) of **3**.

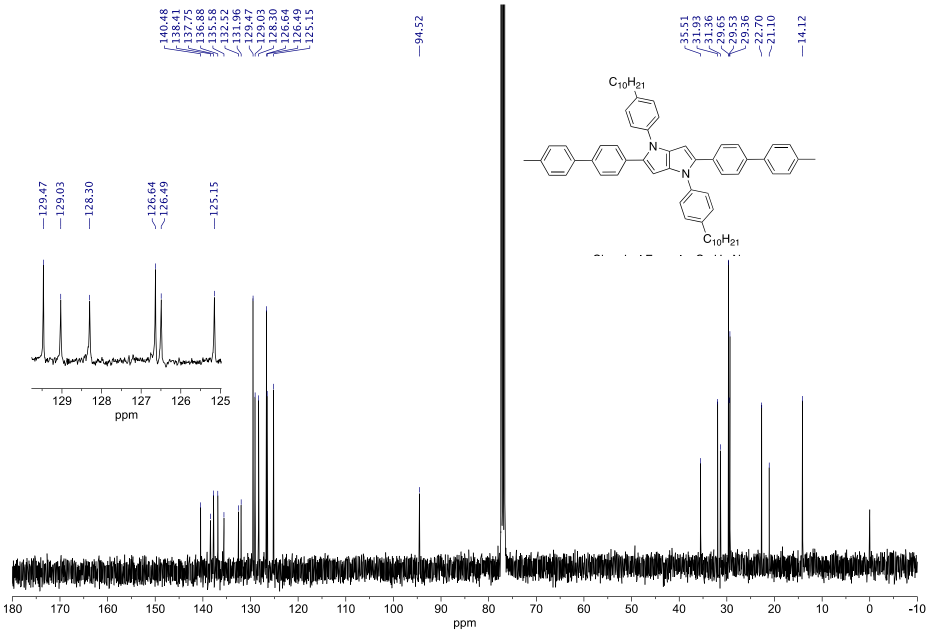

Figure S6. ^{13}C NMR (400 MHz, 25 ˚C, $CDCl_3$) of **3**.

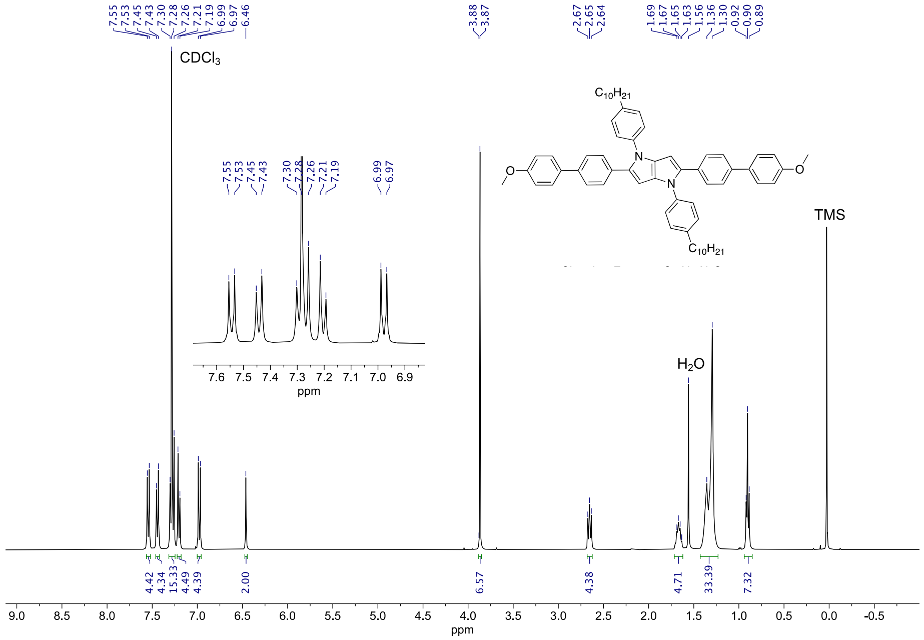

Figure S7. ^{1}H NMR (400 MHz, 25 ˚C, $CDCl_3$) of **4**.

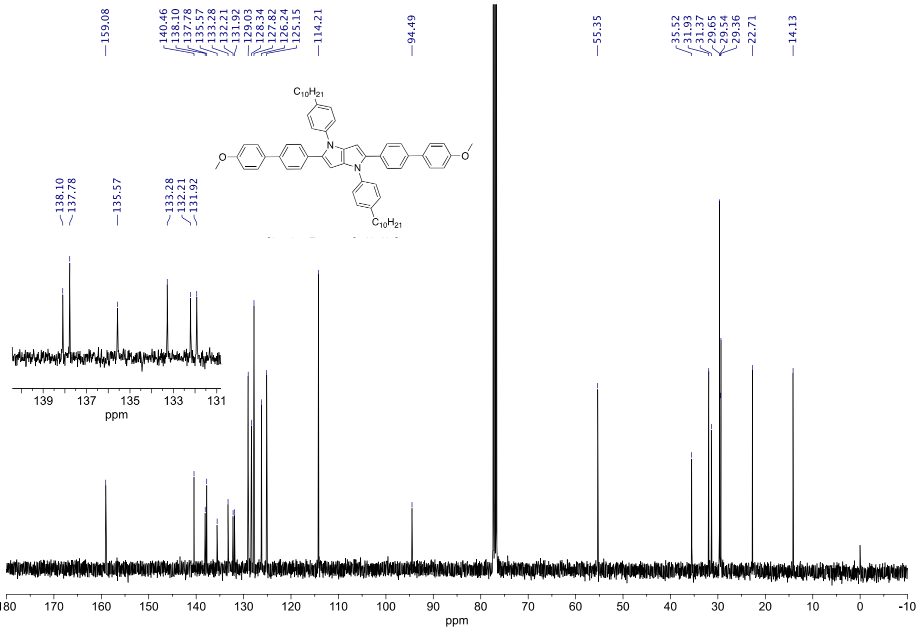

Figure S8. ^{13}C NMR (400 MHz, 25 ˚C, $CDCl_3$) of **4**.

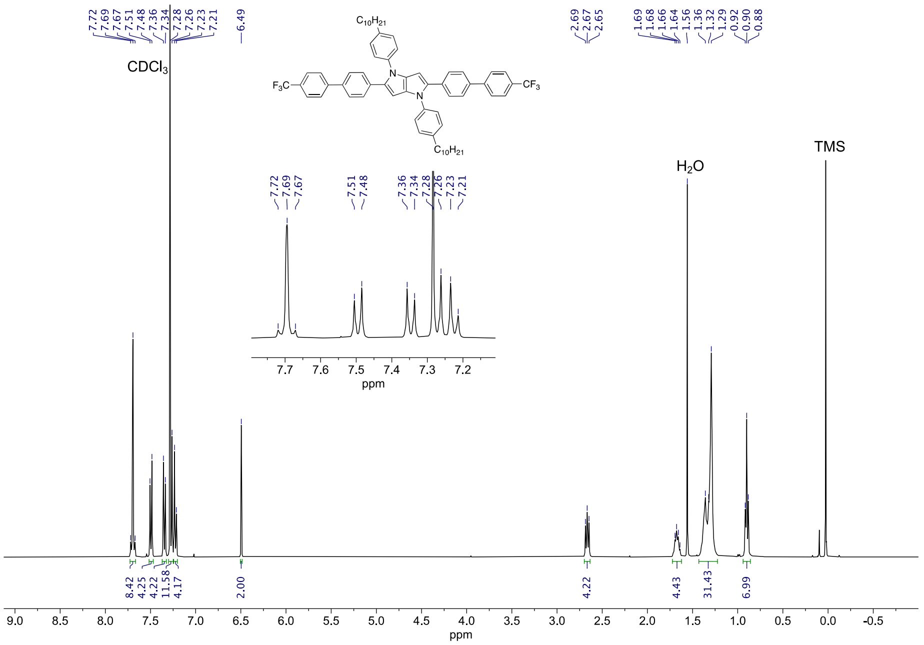

Figure S9. ^{1}H NMR (400 MHz, 25 ˚C, $CDCl_3$) of **5**.

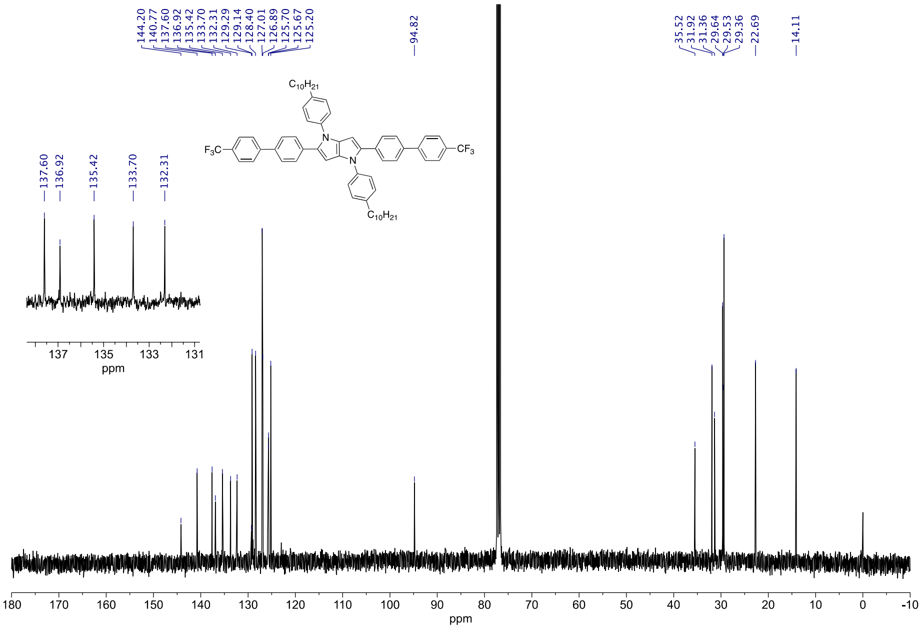

Figure S10. ^{13}C NMR (400 MHz, 25 ˚C, $CDCl_3$) of **5**.

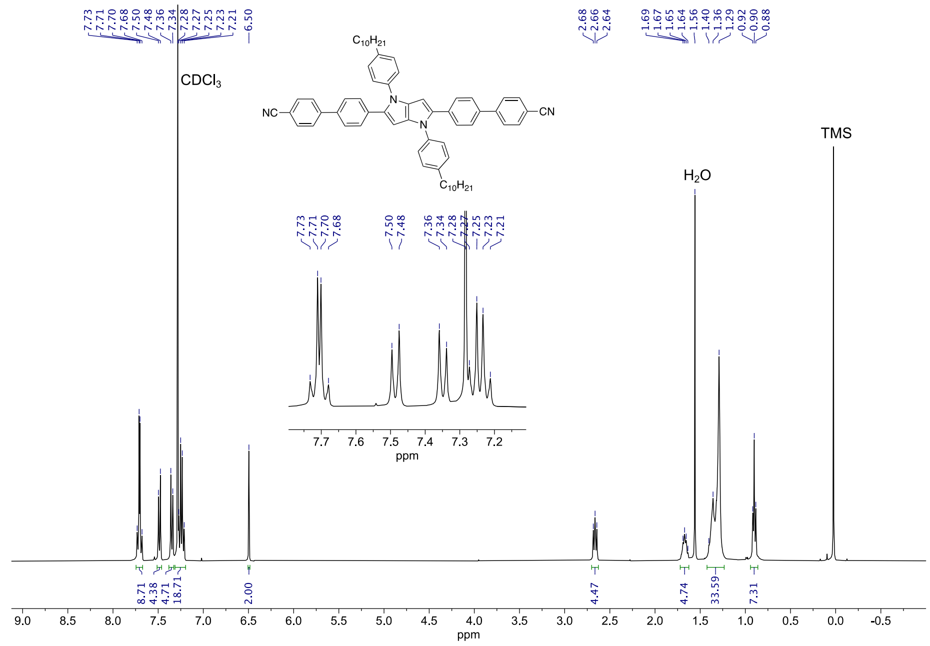

Figure S11. ^{1}H NMR (400 MHz, 25 ˚C, $CDCl_3$) of **6**.

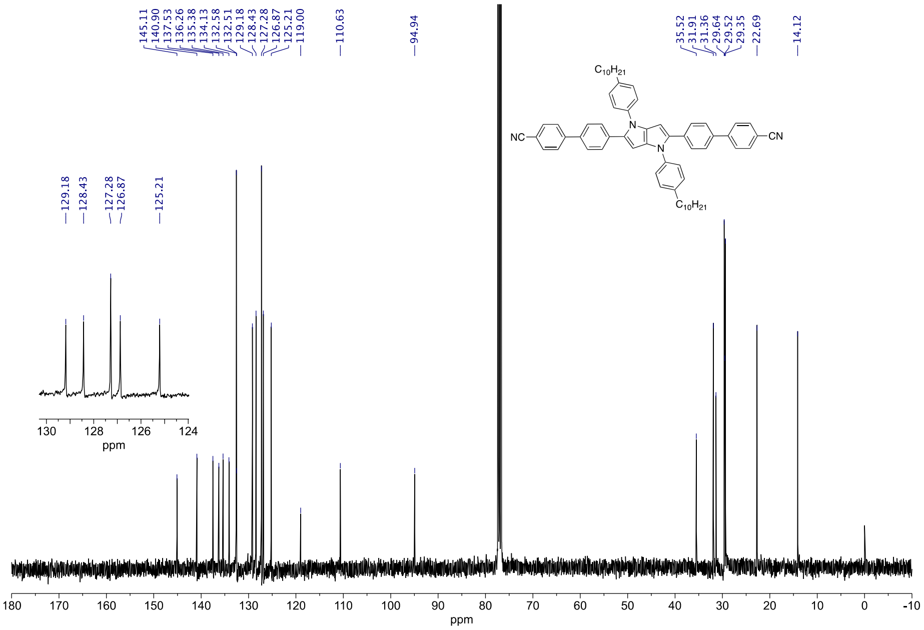

Figure S12. ^{13}C NMR (400 MHz, 25 ˚C, $CDCl_3$) of **6**.

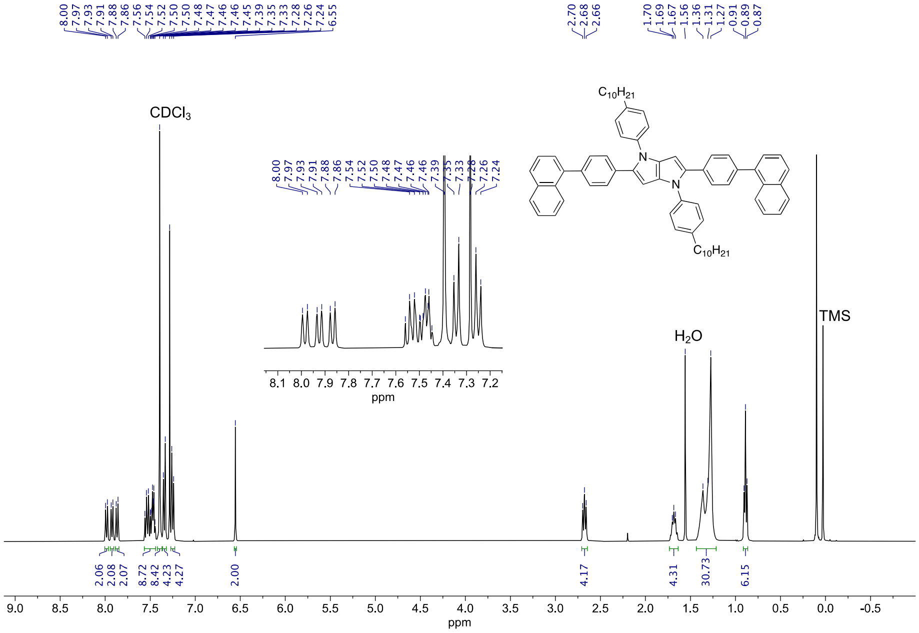

Figure S13. ^{1}H NMR (400 MHz, 25 ˚C, $CDCl_3$) of **7**.

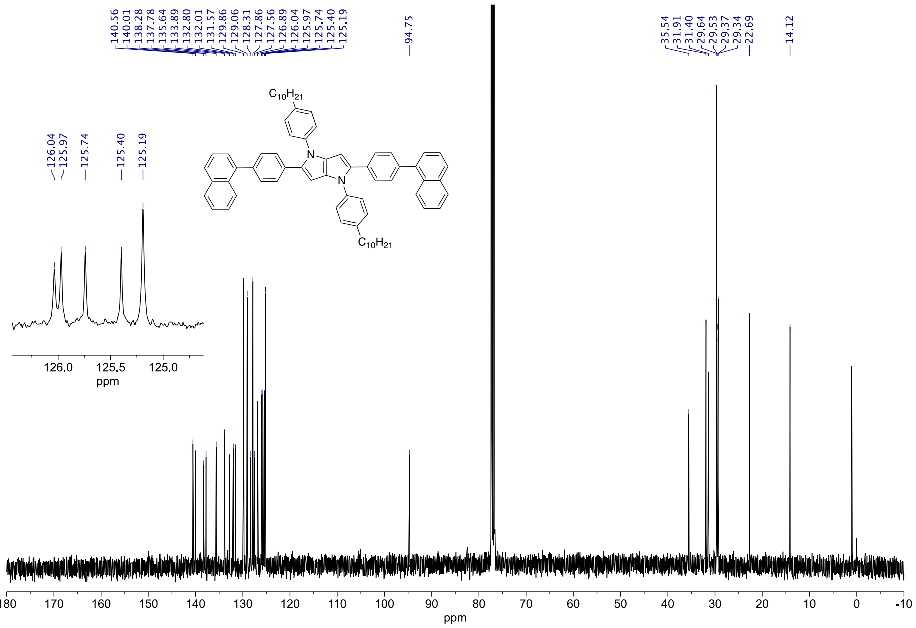

Figure S14. ^{13}C NMR (400 MHz, 25 ˚C, $CDCl_3$) of **7**.

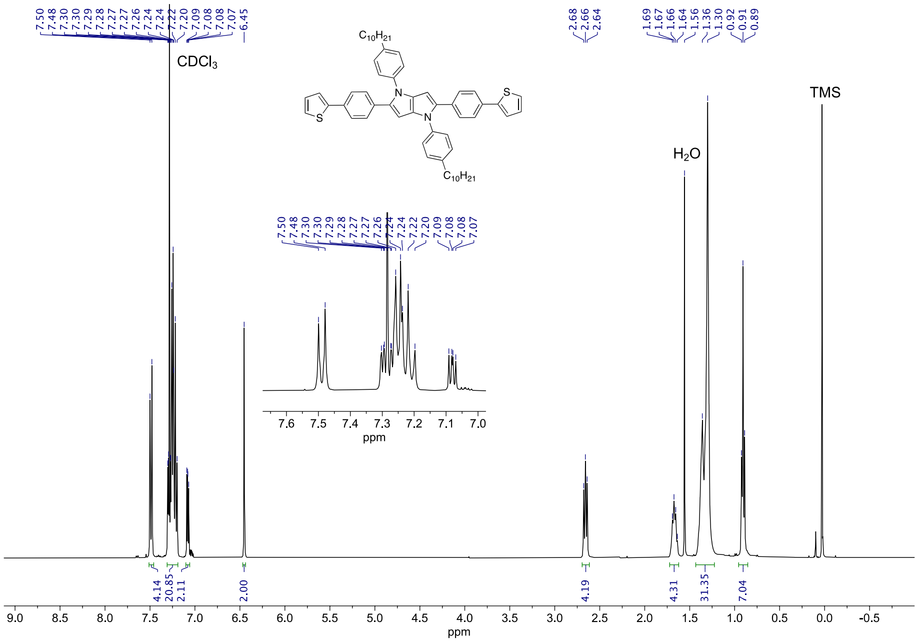

Figure S15. ^{1}H NMR (400 MHz, 25 ˚C, $CDCl_3$) of **8**.

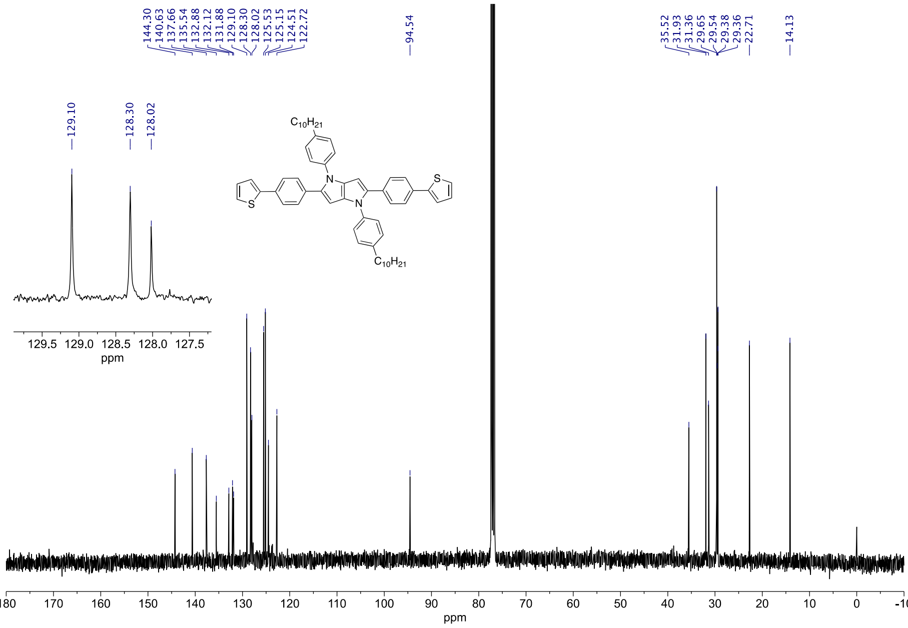

Figure S16. ^{13}C NMR (400 MHz, 25 ˚C, $CDCl_3$) of **8**.

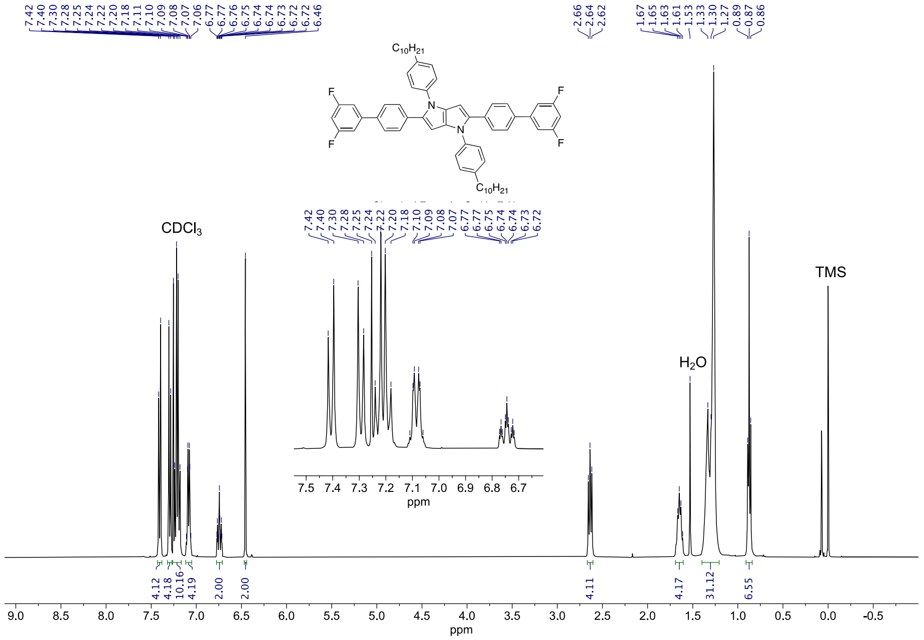

Figure S17. ^{1}H NMR (400 MHz, 25 ˚C, $CDCl_3$) of **9**.

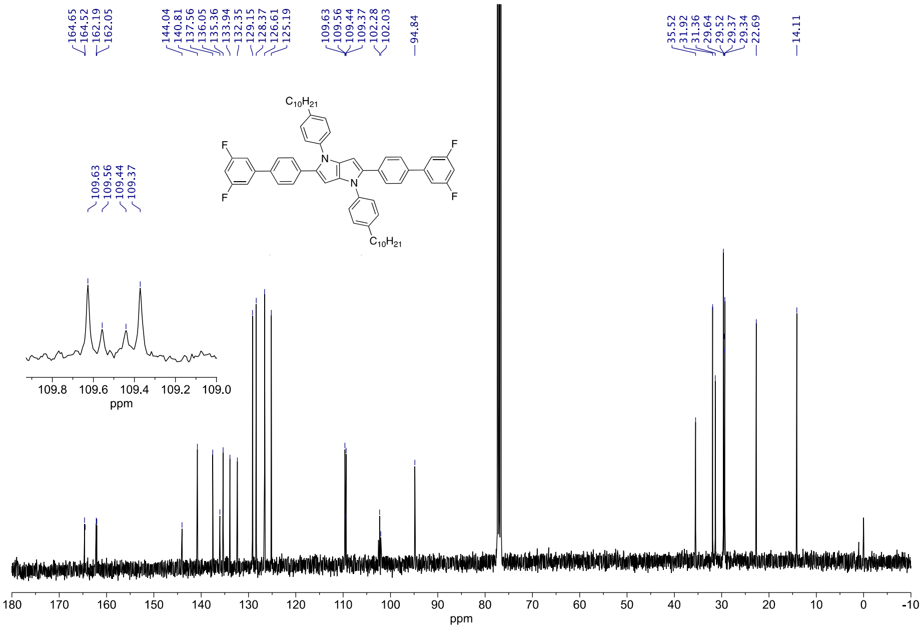

Figure S18. ^{13}C NMR (400 MHz, 25 ˚C, $CDCl_3$) of **9**.

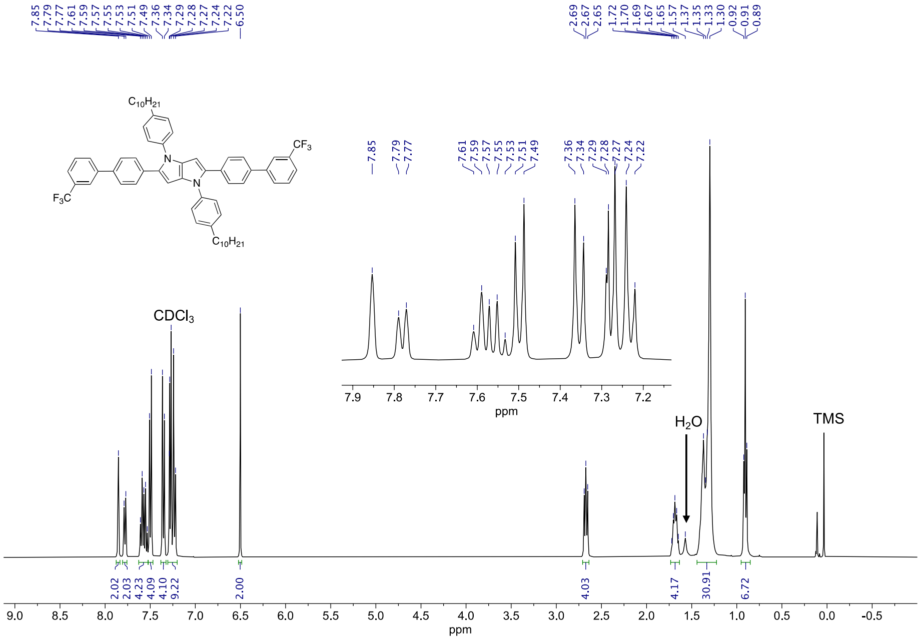

Figure S19. ^{1}H NMR (400 MHz, 25 ˚C, $CDCl_3$) of **10**.

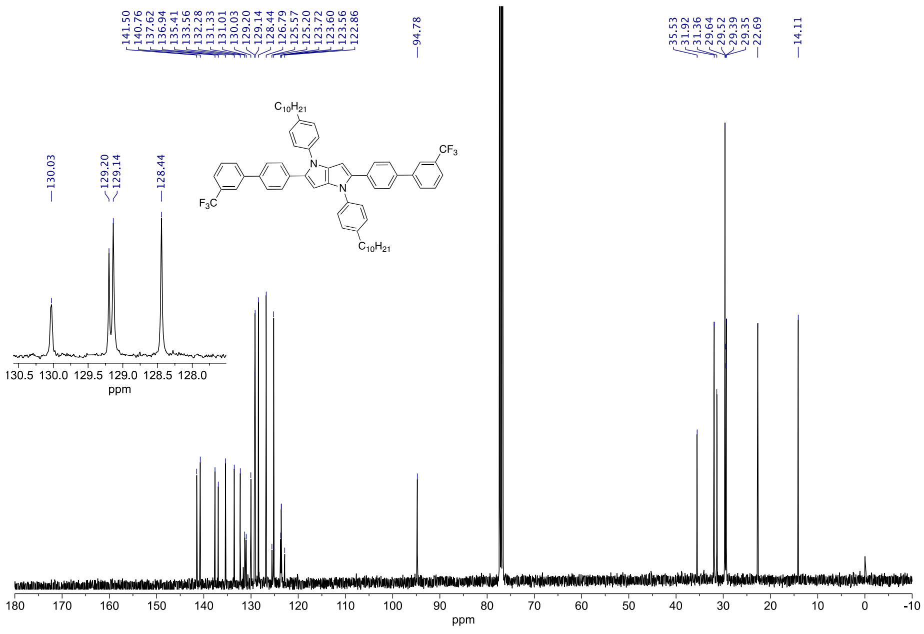

Figure S20. ^{13}C NMR (400 MHz, 25 ˚C, $CDCl_3$) of **10**.

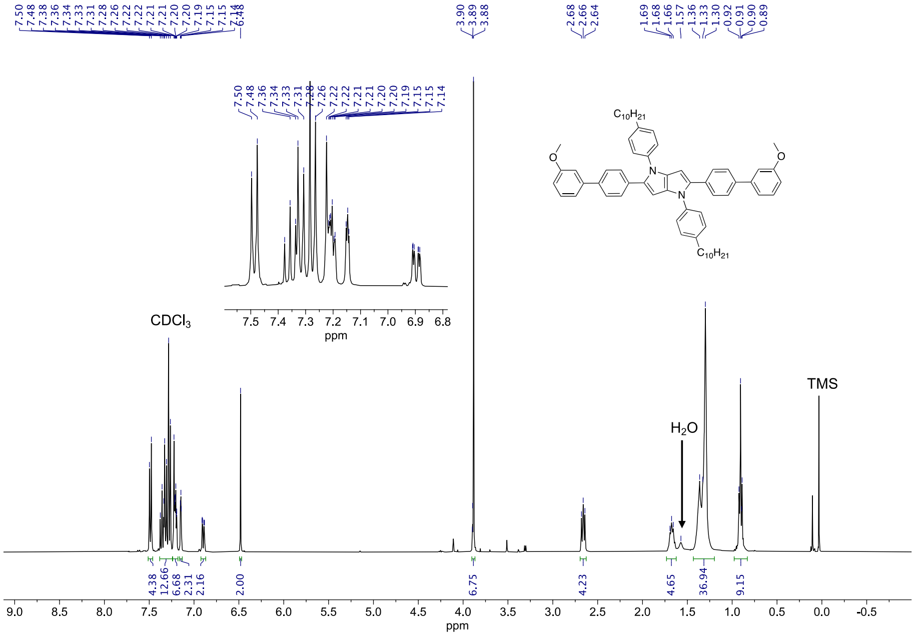

Figure S21. ^{1}H NMR (400 MHz, 25 ˚C, $CDCl_3$) of **11**.

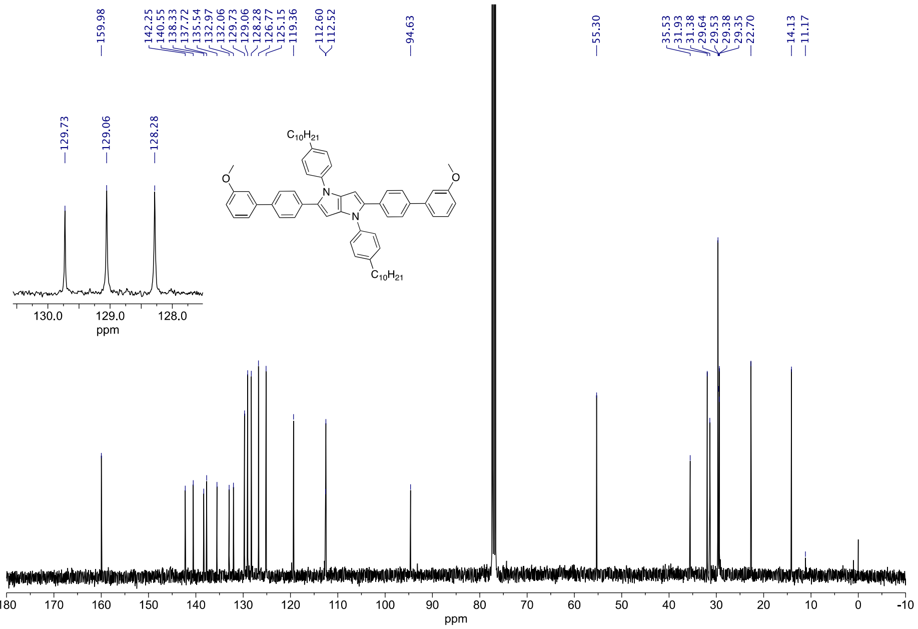

Figure S22. ^{13}C NMR (400 MHz, 25 ˚C, $CDCl_3$) of **11**.

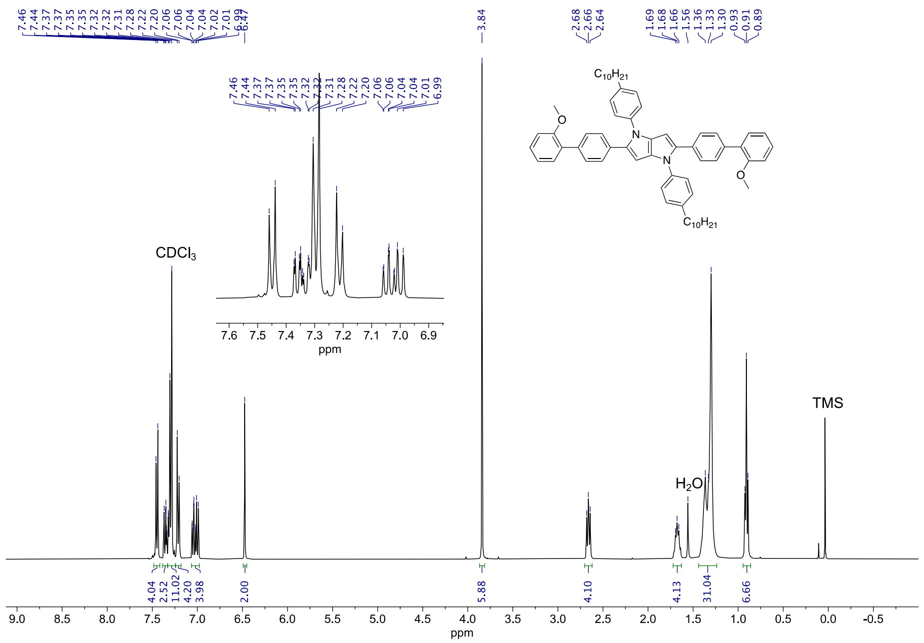

Figure S23. ^{1}H NMR (400 MHz, 25 ˚C, $CDCl_3$) of **12**.

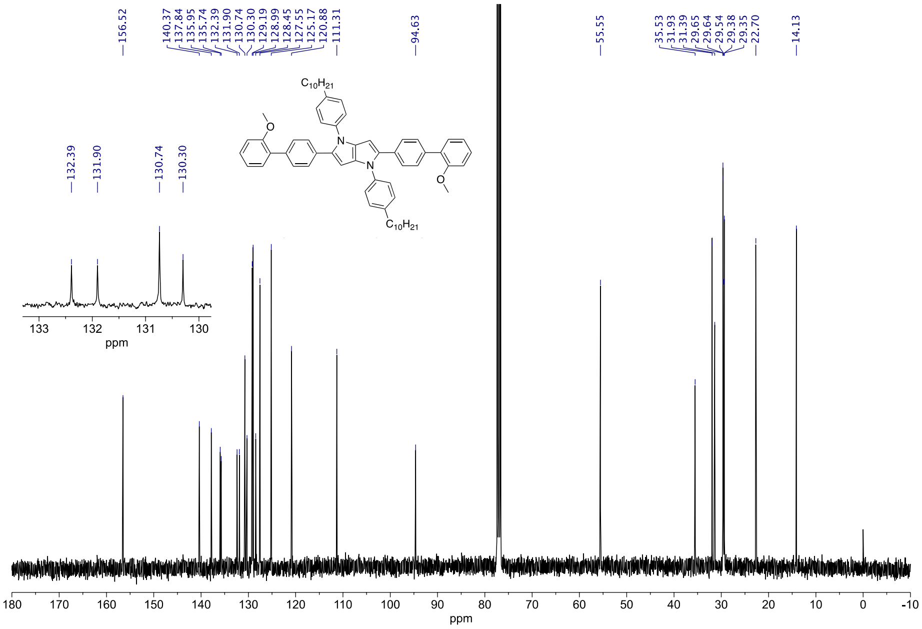

Figure S24. ^{13}C NMR (400 MHz, 25 ˚C, $CDCl_3$) of **12**.

<u>UV-Vis</u>

When studying the optical properties of DHPPs **7-12**, changing the substitution position was found not to alter the optical properties significantly. For example, and as shown in Figure

S25 and Table S2, the methoxy-functionalized DHPPs, DHPP **4** and DHPP **11**, have similar λ_{max} values = 382-383 nm while DHPP **12**, with a methoxy group at the *meta*-position, has a λ_{max} of 378 nm. The blue-shift from DHPP **11** to DHPP **12** may be attributed to increased torsional angles that disrupt the chromophores conjugation by positioning the OMe at the *ortho*-position with respect to the DHPP core.[9,10] The same trend is seen with the CF_3-functionalized coupling partners, with the *para*-functionalized chromophore DHPP **5** having a λ_{max} = 397 nm while the *meta*-functionalized chromophore DHPP **10** has a λ_{max} = 391 nm. The 6 nm difference between the *para*- and *meta*-CF_3 chromophores is likely due to a decreased push-pull effect by removing the electron-withdrawing substituent from the conjugation pathway.[11,12] A multi-fluorinated coupling partner, DHPP **9,** yields a slightly red-shifted λ_{max} = 394 nm compared to DHPP **10** ($\lambda_{max} \approx$ 391 nm) due to the increased electronegativity of the fluorine atoms that increases the push-pull effect. Alternatively, the naphthalene- and thienyl-functionalized DHPPs, DHPP **7** and DHPP **8** respectively, display λ_{max} values of 379 nm and 400 nm (Figure S25B and Table S2). DHPP **7** shows a slightly red-shifted absorbance compared to other PAH-functionalized DHPPs (λ_{max} ~ 369 nm) due to extending the π-conjugation[13,14] while the increased quinoidal character of thiophene, and the subsequent reduction in dihedral angles,[15] results in the red shift observed for DHPP **8** compared to other π-extended DHPPs with electron-donating characteristics. The synthetic efforts represent the robust nature of functionalizing DHPPs through Pd-catalyzed cross-coupling reactions with diverse aromatic functionalities. The structural diversity, in turn, moderately influences the optical properties of the neutral molecules.

Table S2. UV-vis absorbance maxima for the neutral π-extended DHPP chromophores.

**Chromophore**	**λ_{max} Neu (nm)**
**1**	405
**2**	383
**3**	383
**4**	382
**5**	397
**6**	412
**7**	379
**8**	400
**9**	394
**10**	391
**11**	383
**12**	378

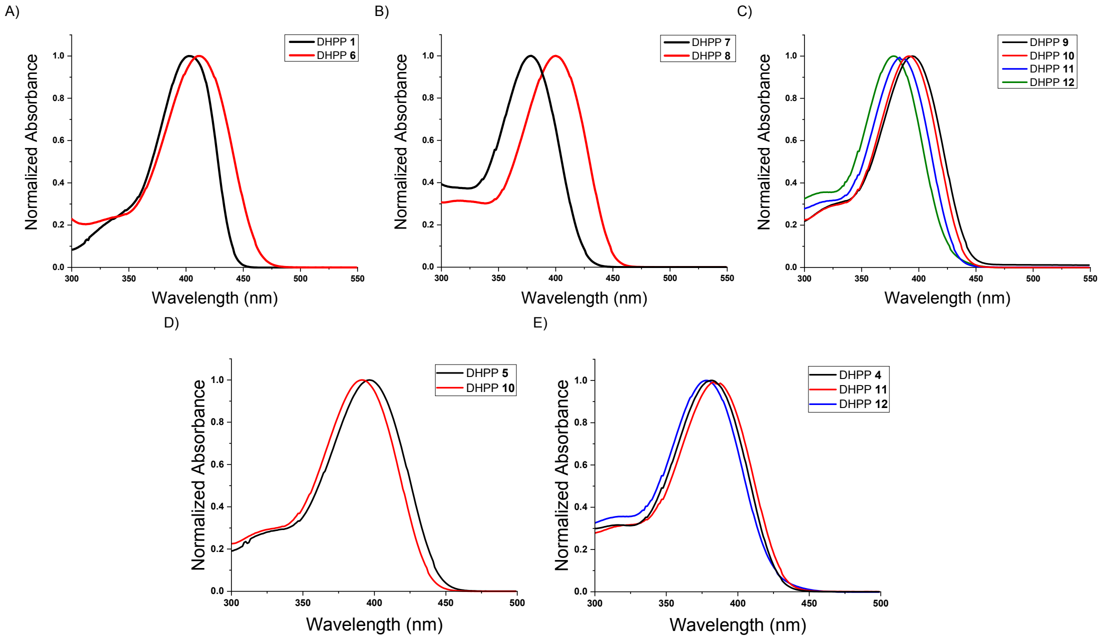

Figure S25. UV-vis absorbance spectra for the varying substituent choice and position of DHPP chromophores.

Electrochemistry

DHPP **1** and DHPP **6** were compared to understand the influence of increased conjugation on the redox activity. As shown in Figure S26A, DHPP **1** has an onset of oxidation ~ 0.68 V which is slightly higher compared to DHPP **6** whose onset of oxidation was measured to be ~ 0.58 V (vs. Ag/AgCl) (Table S3). The lower onset of oxidation from DHPP **1** to DHPP **6** is attributed to the

increased π-conjugation which reduces the voltage required to remove an electron.[16] By increasing the π-conjugation, the redox properties of DHPPs can be manipulated and reinforces this approach as a functional handle for controlling the redox properties of DHPP molecules.

Table S3. Electronic properties of DHPP **1** and DHPP **6** obtained from electrochemical and optical characterizations to understand the influence of π-conjugation on redox activity.

Chromophore	E_{onset}^{ox} (V)	HOMO (eV)[a]	LUMO (eV)[b]	E_{gap} (eV)[c]
**1**	0.68	-5.8	-3.1	2.7
**6**	0.58	-5.7	-3.1	2.6

[a]Calculated given HOMO = - (E_{onset}^{ox} + 5.12 eV); [b]Calculated from absorbance onset given eV = $1240/\lambda_{onset}$ + HOMO; [c]Calculated from (LUMO – HOMO); all equations are adopted from Cardona and coworkers.[17]

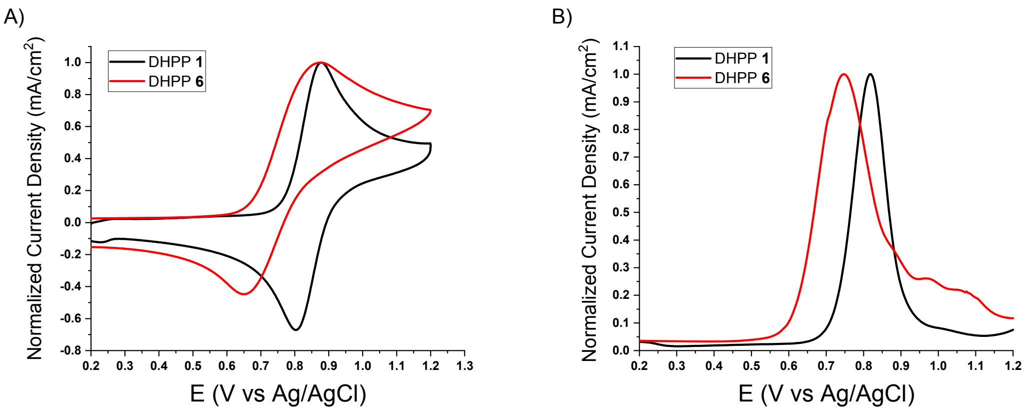

Figure S26. Comparison of redox response of DHPP **1** and DHPP **6** using (A) CV and (B) DPV in a 0.5 M $TBAPF_6$/DCM supporting electrolyte against Fc/Fc^+ calibration illustrating the effect of increasing the π-conjugation on the redox activity of DHPPs.

<u>Solution Oxidation Studies</u>

The fundamental comparison of how the increased conjugation length influences radical cation properties was addressed first by comparing changes in the absorbance of DHPP **1** and DHPP **6**. Figure S27 displays the transition of the neutral UV-vis absorbance spectra to the absorbance spectra of the molecules as they are oxidized. Upon oxidization, DHPP **1** and DHPP **6** shift further into the visible region of the EMS and display characteristics of SOMO-α → LUMO-

α and SOMO-β → LUMO-β transitions.[18–22] For DHPP **1**, the SOMO-α → LUMO-α at 465 nm is broad and less prominent than the same transition associated with DHPP **6** (490 nm) which may be attributed to the radical cation of DHPP **1** having a smaller molar absorptivity compared to the radical cation of DHPP **6**. The red shift in the maximum absorbance for the SOMO-α → LUMO-α ($\lambda^{\alpha}_{\mathrm{max}}$) from DHPP **1** to DHPP **6** is attributed to the increase in the conjugation length (Table S4). Going from the neutral absorbance to the SOMO-α → LUMO-α, DHPP **6** has a more exacerbated red shift with an ~ 80 nm shift while DHPP **1** only has a ~ 60 nm shift. The SOMO-β transitions follow the same trend as the SOMO-α transitions where absorbance maximum for the SOMO-β → LUMO-β (λ^{β}_{max}) of DHPP **1** is located ~ 655 nm and the λ^{β}_{max} of DHPP **6** ~ 730 nm. There also is a difference in the absorbance profile of the SOMO-β → LUMO-β with DHPP **1** having a lower intensity at ~ 0.2 absorbance units while DHPP **6** has a more intense SOMO-β transition. The more intense absorbance for DHPP **6** is attributed to both the difference in molar absorptivity and the increased rotational freedom of the π-extended system, which enables more allowed excited state geometries.[23] Due to the differences in the λ_{max} and absorbance profiles of the SOMO → LUMO transitions, it can be determined that through the manipulation of the π-conjugation of DHPP chromophores, control of the absorbance profiles is achieved.

Table S4. UV-vis and color coordinate data for DHPP **1** and DHPP **6**. The neutral and oxidized λ_{max} values correspond to the SOMO-α → LUMO-α and SOMO-β → LUMO-β, while the neutral and oxidized color coordinates are calculated based on mid-day lighting standards (D50 illuminant as a 2° observer).

**Chromophore**	λ^{max}_{neu} (nm)	λ^{max}_{ox} (nm)		**Color Coordinates**	
		**SOMO-α**	**SOMO-β**	**Neu. (*L*a*b**)**	**Ox. (*L*a*b**)**
**1**	405	465	655	100, -5, 12	93, -14, 39
**6**	412	490	730	100, -11, 37	83, 10, 52

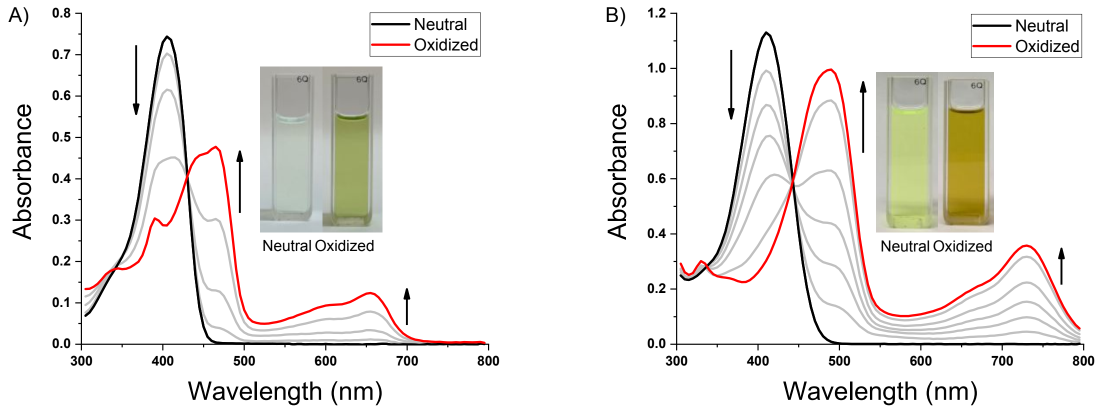

Figure S27. The solution oxidation spectra of (A) DHPP **1** and (B) DHPP **6** in DCM using 0.06 mg/mL $Fe(ClO_4)_3$□xH_2O in ethyl acetate as the dopant. These spectra display changes in the UV-vis absorbance spectra with increasing dopant concentration to elucidate and compare the ability of DHPPs to be chemically doped.

<u>Colorimetry</u>

For DHPP **1**, the neutral solution was found to have color coordinates of 100, -5, 12 (Table S4). Within the coordinate diagram, these values correspond to a decreased saturation level and the solution being perceived as nearly colorless or transmissive (Figure S28). The π-extended DHPP **6** experiences color coordinates of 100, -11, 37, which is a light-yellow color when placed on the coordinate diagram and is consistent with the red-shifted UV-vis data reported in Figure 4 (Figure 6). This coloration is confirmed by the photographs within the insets of Figure S28. Upon oxidation, both DHPPs display a shift in their solution color, resulting in *L*a*b** color coordinates of 93, -14, 39 for DHPP **1** and 83, 10, 52 for DHPP **6**. The color transitions for these two DHPPs are transmissive-to-yellow-green for DHPP **1,** while DHPP **6** exhibits a yellow-to-golden-yellow color change. The green color arises from the dual-band absorbance within the visible region of the EMS with a transmissive window from ~ 500-550 nm measured via UV-vis for the oxidized DHPP **1**.[24] The red shift from DHPP **1** to DHPP **6** illustrated in the UV-vis absorbance spectra is further exemplified here, with the location of the colors of the oxidized chromophores being in

two separate quadrants. The differences in the color profile for the two DHPPs and the ability to fine-tune the radical cation absorbance through the conjugation length motivate the continued investigation into how the coupling partner impacts the radical cation of DHPPs and ultimately color of oxidized solutions.

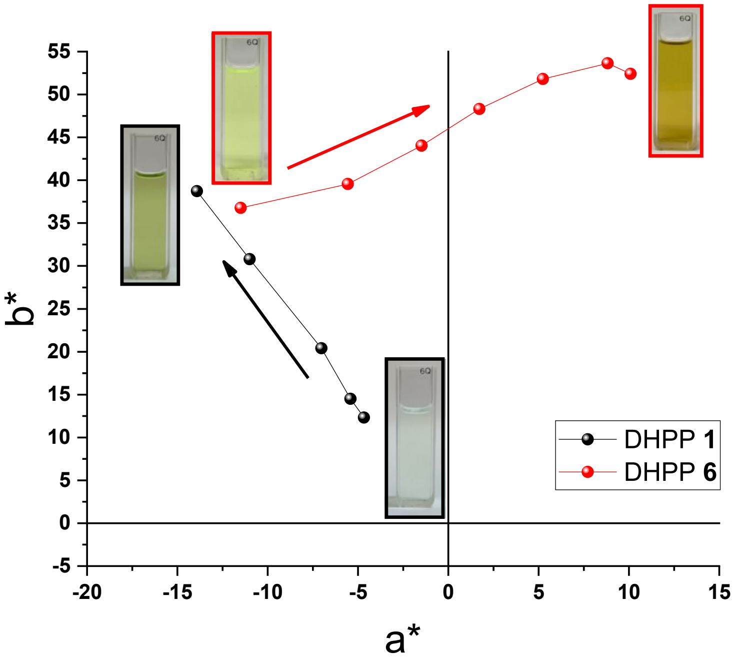

Figure S28. Color coordinate data for DHPP **1** and DHPP **6** obtained as a function of dopant concentration that quantify the color difference experienced by the π-extended systems.

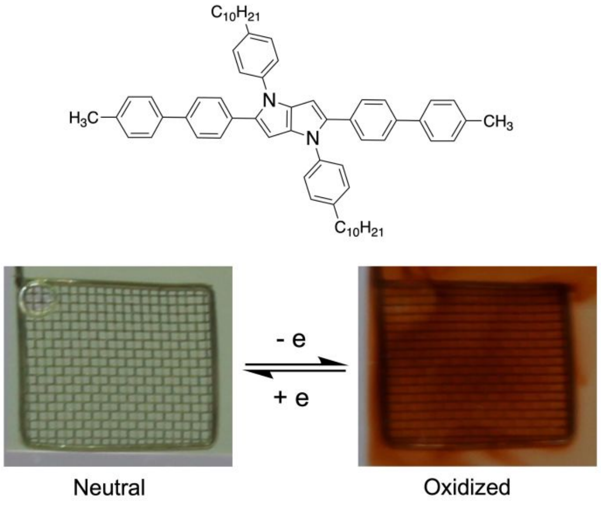

Figure S29. Electrochemical switching experiments using an OTTLE of the color-controlled, high-contrast DHPP chromophore DHPP **3**.

<u>References</u>

(1) Bell, K.-J. J.; Kisiel, A. M.; Smith, E.; Tomlinson, A. L.; Collier, G. S. Simple Synthesis of Conjugated Polymers Enabled via Pyrrolo[3,2-*b*]Pyrroles. *Chem. Mater.* **2022**, *34*, 8729–8739. https://doi.org/10.1021/acs.chemmater.2c01884.

(2) Domínguez, R.; Montcada, N. F.; de la Cruz, P.; Palomares, E.; Langa, F. Pyrrolo[3,2-*b*]Pyrrole as the Central Core of the Electron Donor for Solution-Processed Organic Solar Cells. *Chempluschem* **2017**, *82* (7), 1096–1104. https://doi.org/10.1002/cplu.201700158.

(3) Tasior, M.; Clermont, G.; Blanchard-Desce, M.; Jacquemin, D.; Gryko, D. T. Synthesis of Bis(Arylethynyl)Pyrrolo[3,2-*b*]Pyrroles and Effect of Intramolecular Charge Transfer on Their Photophysical Behavior. *Chem. Eur. J.* **2019**, *25* (2), 598–608. https://doi.org/10.1002/chem.201804325.

(4) Stezycki, R.; Reger, D.; Hoelzel, H.; Jux, N.; Gryko, D. T. Synthesis and Photophysical Properties of Hexaphenylbenzene-Pyrrolo[3,2-*b*]Pyrroles. *Synlett* **2018**, *29* (19), 2529–2534. https://doi.org/10.1055/s-0037-1610286.

(5) Krzeszewski, M.; Gryko, D.; Gryko, D. T. The Tetraarylpyrrolo[3,2-*b*]Pyrroles - From Serendipitous Discovery to Promising Heterocyclic Optoelectronic Materials. *Acc. Chem. Res.* **2017**, *50* (9), 2334–2345. https://doi.org/10.1021/acs.accounts.7b00275.

(6) Allen, E. The Melting Point of Impure Organic Compounds. *J. Chem. Educ.* **1942**, *19* (6), 278–281. https://doi.org/10.1021/ed019p278.

(7) Brown, R. J. C.; Brown, R. F. C. Melting Point and Molecular Symmetry. *J. Chem. Educ.* **2000**, *77* (6), 724–731. https://doi.org/10.1021/ed077p724.

(8) Janiga, A.; Krzeszewski, M.; Gryko, D. T. Diindolo[2,3-*b*:2′,3′-*f*]Pyrrolo[3,2-*b*]Pyrroles as Electron-Rich, Ladder-Type Fluorophores: Synthesis and Optical Properties. *Chem. - An*

Asian J. **2015**, *10* (1), 212–218. https://doi.org/10.1002/asia.201402925.

(9) Liu, X.; Xu, Z.; Cole, J. M. Molecular Design of UV-Vis Absorption and Emission Properties in Organic Fluorophores: Toward Larger Bathochromic Shifts, Enhanced Molar Extinction Coefficients, and Greater Stokes Shifts. *J. Phys. Chem. C* **2013**, *117* (32), 16584–16595. https://doi.org/10.1021/jp404170w.

(10) Jin, X.; Li, S.; Guo, L.; Hua, J.; Qu, D. H.; Su, J.; Zhang, Z.; Tian, H. Interplay of Steric Effects and Aromaticity Reversals to Expand the Structural/Electronic Responses of Dihydrophenazines. *J. Am. Chem. Soc.* **2022**, *144* (11), 4883–4896. https://doi.org/10.1021/jacs.1c12610.

(11) Jiang, Y.; Cabanetos, C.; Allain, M.; Liu, P.; Roncali, J. Manipulation of the Band Gap and Efficiency of a Minimalist Push-Pull Molecular Donor for Organic Solar Cells. *J. Mater. Chem. C* **2015**, *3* (20), 5145–5151. https://doi.org/10.1039/c5tc00913h.

(12) Irfan, A.; Aftab, H.; Al-Sehemi, A. G. Push-Pull Effect on the Geometries, Electronic and Optical Properties of Thiophene Based Dye-Sensitized Solar Cell Materials. *J. Saudi Chem. Soc.* **2014**, *18* (6), 914–919. https://doi.org/10.1016/j.jscs.2011.11.013.

(13) Banasiewicz, M.; Stężycki, R.; Kumar, G. D.; Krzeszewski, M.; Tasior, M.; Koszarna, B.; Janiga, A.; Vakuliuk, O.; Sadowski, B.; Gryko, D. T.; et al. Electronic Communication in Pyrrolo[3,2-*b*]Pyrroles Possessing Sterically Hindered Aromatic Substituents. *Euro J. Org. Chem.* **2019**, 5247–5253. https://doi.org/10.1002/ejoc.201801809.

(14) Tasior, M.; Koszarna, B.; Young, D. C.; Bernard, B.; Jacquemin, D.; Gryko, D.; Gryko, D. T. Fe(III)-Catalyzed Synthesis of Pyrrolo[3,2-*b*]Pyrroles: Formation of New Dyes and Photophysical Studies. *Org. Chem. Front.* **2019**, *6* (16), 2939–2948. https://doi.org/10.1039/c9qo00675c.

(15) Zhang, Z. G.; Wang, J. Structures and Properties of Conjugated Donor-Acceptor Copolymers for Solar Cell Applications. *J. Mater. Chem.* **2012**, *22* (10), 4178–4187. https://doi.org/10.1039/c2jm14951f.

(16) Hanson, K.; Roskop, L.; Djurovich, P. I.; Zahariev, F.; Gordon, M. S.; Thompson, M. E. A Paradigm for Blue- or Red-Shifted Absorption of Small Molecules Depending on the Site of π-Extension. *J. Am. Chem. Soc.* **2010**, *132* (45), 16247–16255. https://doi.org/10.1021/ja1075162.

(17) Cardona, C. M.; Li, W.; Kaifer, A. E.; Stockdale, D.; Bazan, G. C. Electrochemical Considerations for Determining Absolute Frontier Orbital Energy Levels of Conjugated Polymers for Solar Cell Applications. *Adv. Mater.* **2011**, *23* (20), 2367–2371. https://doi.org/10.1002/adma.201004554.

(18) Nhon, L.; Tennyson, S. L.; Butt, M. W.; Bacsa, J.; Tomlinson, L.; Reynolds, J. R. Theory-Driven Spectral Control of Bis-EDOT Arylene Radical Cation Chromophores. *Chem. Mater.* **2022**, *34* (21), 9546–9557. https://doi.org/10.1021/acs.chemmater.2c02054.

(19) Nhon, L.; Wilkins, R.; Reynolds, J. R.; Tomlinson, A. Guiding Synthetic Targets of Anodically Coloring Electrochromes through Density Functional Theory. *J. Chem. Phys.* **2021**, *154*, 054110. https://doi.org/10.1063/5.0039511.

(20) Christiansen, D. T.; Tomlinson, A. L.; Reynolds, J. R. New Design Paradigm for Color Control in Anodically Coloring Electrochromic Molecules. *J. Am. Chem. Soc.* **2019**, *141*, 3859–3862. https://doi.org/10.1021/jacs.9b01507.

(21) Österholm, A. M.; Nhon, L.; Shen, D. E.; Dejneka, A. M.; Tomlinson, A. L.; Reynolds, J. R. Conquering Residual Light Absorption in the Transmissive States of Organic Electrochromic Materials. *Mater. Horizons* **2022**, *9* (1), 252–260.

https://doi.org/10.1039/d1mh01136g.

(22) Li, G.; Song, R.; Ma, W.; Liu, X.; Li, Y.; Rao, B.; He, G. π-Extended Chalcogenoviologens with Stable Radical State Enable Enhanced Visible-Light-Driven Hydrogen Evolution and Static/Dynamic Electrochromic Displays. *J. Mater. Chem. A* **2020**, *8* (25), 12278–12284. https://doi.org/10.1039/d0ta02930k.

(23) Gu, Q.; Chotard, F.; Eng, J.; Reponen, A. P. M.; Vitorica-Yrezabal, I. J.; Woodward, A. W.; Penfold, T. J.; Credgington, D.; Bochmann, M.; Romanov, A. S. Excited-State Lifetime Modulation by Twisted and Tilted Molecular Design in Carbene-Metal-Amide Photoemitters. *Chem. Mater.* **2022**, *34* (16), 7526–7542. https://doi.org/10.1021/acs.chemmater.2c01938.

(24) Beaujuge, P. M.; Amb, C. M.; Reynolds, J. R. Spectral Engineering in π-Conjugated Polymers with Intramolecular Donor-Acceptor Interactions. *Acc. Chem. Res.* **2010**, *43* (11), 1396–1407. https://doi.org/10.1021/ar100043u.
